# Room temperature ionic liquids with two symmetric ions†

**DOI:** 10.1039/d3sc03240j

**Published:** 2023-09-08

**Authors:** Daniel Rauber, Frederik Philippi, Daniel Schroeder, Bernd Morgenstern, Andrew J. P. White, Marlon Jochum, Tom Welton, Christopher W. M. Kay

**Affiliations:** a Department of Chemistry, Saarland University Campus B 2.2 66123 Saarbrücken Germany daniel.rauber@uni-saarland.de; b Department of Chemistry, Molecular Sciences Research Hub, Imperial College London, White City Campus London W12 0BZ UK f.philippi18@imperial.ac.uk; c INM-Leibniz Institute for New Materials Campus D2.2 66123 Saarbrücken Germany; d London Centre for Nanotechnology, University College London 17-19 Gordon Street London WC1H 0AH UK c.kay@ucl.ac.uk

## Abstract

Room temperature ionic liquids typically contain asymmetric organic cations. The asymmetry is thought to enhance disorder, thereby providing an entropic counter-balance to the strong, enthalpic, ionic interactions, and leading, therefore, to lower melting points. Unfortunately, the synthesis and purification of such asymmetric cations is typically more demanding. Here we introduce novel room temperature ionic liquids in which both cation and anion are formally symmetric. The chemical basis for this unprecedented behaviour is the incorporation of ether-containing side chains – which increase the configurational entropy – in the cation. Molecular dynamics simulations indicate that the ether-containing side chains transiently sample curled configurations. Our results contradict the long-standing paradigm that at least one asymmetric ion is required for ionic liquids to be molten at room temperature, and hence open up new and simpler design pathways for these remarkable materials.

## Abstract 2

Here we present ionic liquids composed of highly symmetric cations and anions. The ionic liquids show competitive physical properties despite the high symmetry. The observed behaviour is rationalised with the concept of configurational entropy.

## Graphical abstract

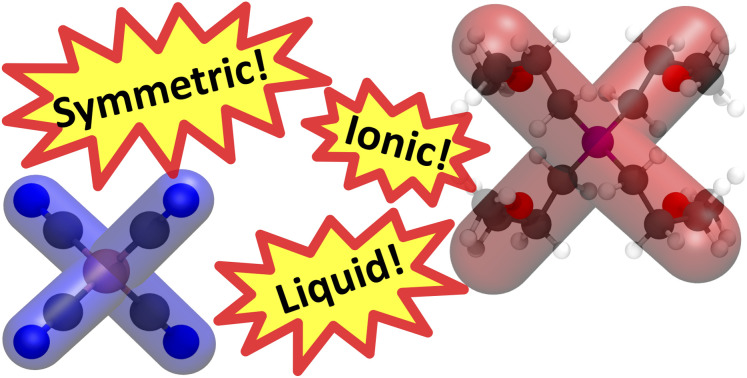

## Introduction

At this point in time, most active researchers in chemistry and its adjacent fields will have heard of ionic liquids. Over the last decades, the use of ionic liquids in academia and industry has increased dramatically.^1–3^ Recent promising examples include biomass treatment and the PETRONAS process for the removal of Hg from natural gas.^4,5^

Ionic liquids are, following the most literal definition, liquids composed entirely of ions. This includes conventional molten salts at high temperature. However, for most purposes, such high temperatures are impractical. The success of ionic liquids is thus coupled to an ongoing hunt for low melting point materials, ideally at or below room temperature (so-called room temperature ionic liquids). From a design point of view, such low melting points can be achieved through a stabilisation of the liquid phase and/or a destabilisation of the crystal.^3,6^ Thermodynamically, the melting point *T*_M_ depends on both the enthalpy of melting (=fusion) Δ*H*_M_ and the entropy of melting Δ*S*_M_, eqn (1).1
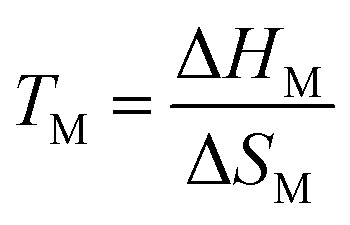


Thus, both enthalpic and entropic contributions from liquid and crystal need to be considered in the design of low melting ionic liquids.^7,8^ Conventional wisdom says that asymmetric ions are crucial in achieving low melting points, and that at least one of the ions needs to be asymmetric to achieve ionic liquids that are molten at room temperature.^3,9,10^ At present, the only feasible way to achieve room temperature ionic liquids with symmetric ions is to use relatively large ions with long alkyl chains, leading to waxy or oily substances with low melting points.^9,11,12^ This approach focuses on the (in)stability of the crystal.

In a recent seminal study, Endo *et al.* demonstrated the importance of configurational entropy.^13^ Briefly, configurational entropy refers to the arrangement of ions in the bulk liquid and has been recognised as a driver for glass formation, supercooling, and fast dynamics.^14–16^ The configurational entropy is high when a large number of microscopic states is accessible, *i.e.* when the potential energy surface contains a large number of accessible basins (many possible configurations) and is flat (minimal energy barriers to changes between configuration).^17,18^ On the other hand, a potential energy surface with only a few well defined basins corresponds to a low configurational entropy. This second situation is usually observed in ionic liquids due to the pronounced charge ordering.

Recent work on ether-functionalised ionic liquids showed that flexibility of the ether group is not the main factor responsible for the high fluidity and low melting points of this type of ionic liquid. Instead, a shift towards a curled cation conformation could be identified as underlying cause.^19–29^ The irregular dependence of physicochemical properties such as density and viscosity on the position of the ether oxygen in the side chain can be rationalised with this shift in conformation.^19,30,31^

**Fig. 1:**
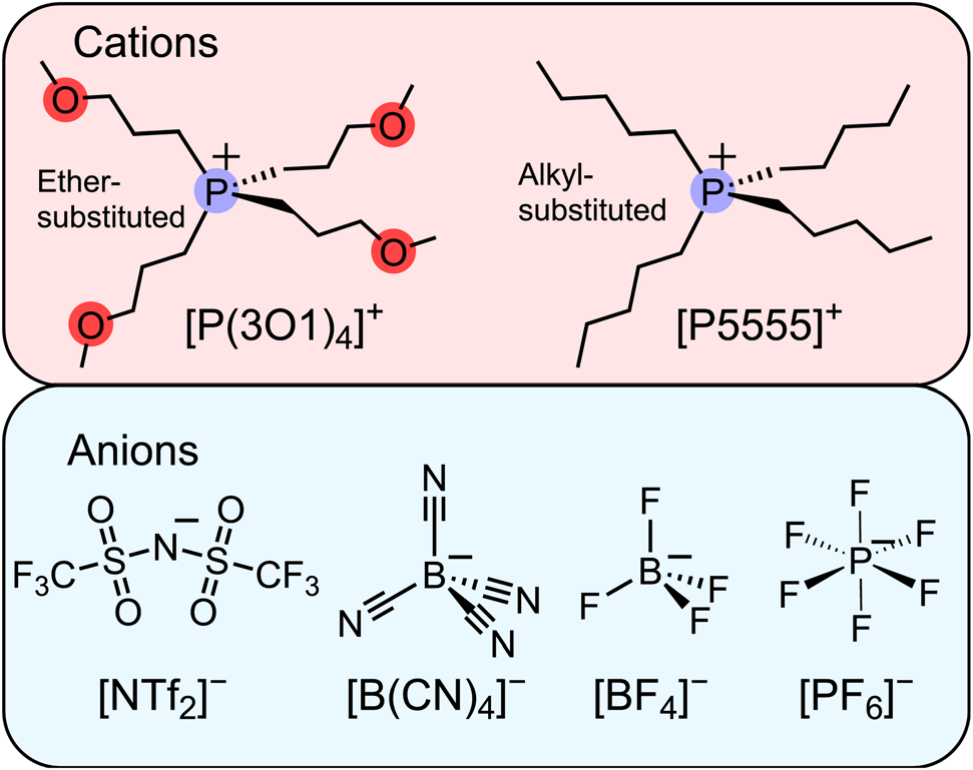
Molecular structures and abbreviations of the symmetric cations and anions forming the ionic liquids investigated in this work. The abbreviations are also defined in the ESI.†

To date, we have followed the common approach of using asymmetric ions as much as possible without further scrutiny. However, our observations from the work with ether-functionalised ionic liquids together with the recent publication by Endo *et al.* led us to consider relatively small, highly symmetric ions as candidates for ionic liquids, Fig. 1. Our hypothesis was that the fourfold ether functionalised cation will introduce significant configurational entropy, enough to lead to room temperature ionic liquids, even when paired with highly symmetric anions. The results we present in this work demonstrate that even the combination of two small ions can lead to room temperature ionic liquids. The performance of these ionic liquids surpassed our expectations and provides inspiration for future studies beyond the current paradigms.

## Results and discussion

### Enthalpy and entropy of melting

**Fig. 2:**
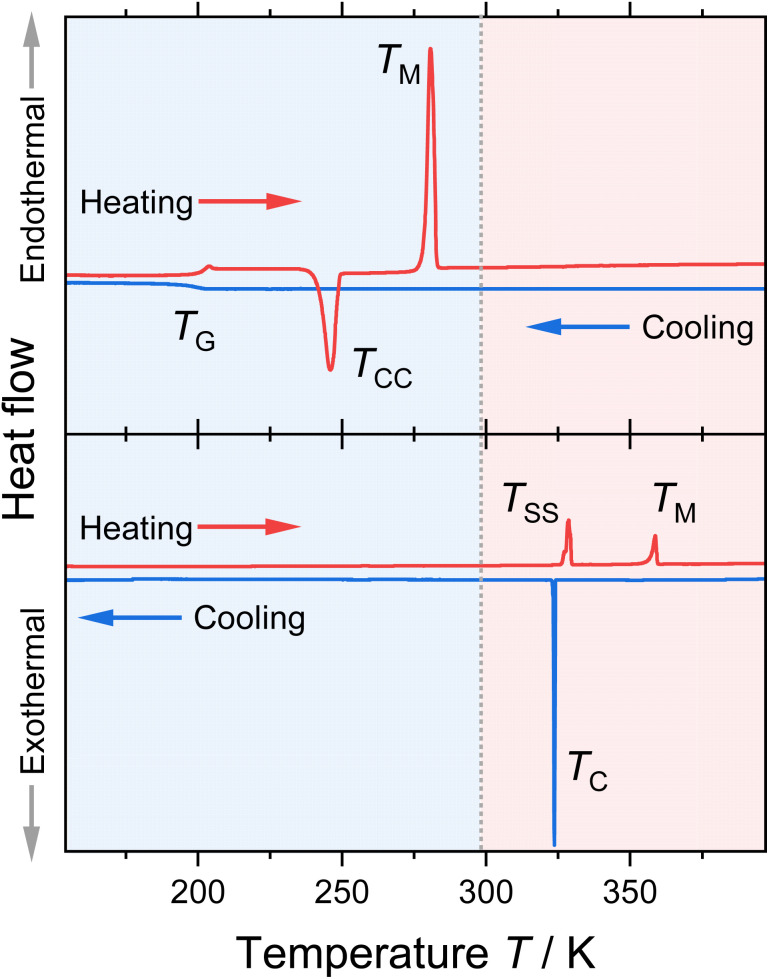
Heating and cooling traces of [P(3O1)_4_][BF_4_] (upper graph) and [P5555][BF_4_] (lower graph) obtained by DSC. The obtained thermal transition points are indicated in the graphs (*T*_C_: crystallisation, *T*_G_: glass transition, *T*_M_: melting; *T*_CC_: cold-crystallisation; *T*_SS_: solid–solid transition). The grey dotted line at 298 K marks the melting point limit for room-temperature ionic liquids.

The thermal behaviour for all combinations with the two cations and five anions was determined by differential scanning calorimetry (DSC). The results are given in Table 1; exemplary DSC traces of the [BF_4_]^−^ samples are shown in Fig. 2. The traces for the other ionic liquids and the obtained thermal transitions with the corresponding enthalpies are given in the ESI.†

**Table 1:** Thermal transitions (*T*_C_: crystallisation, *T*_G_: glass transition, *T*_M_: melting) of the investigated ionic liquids including enthalpy Δ*H*_M_ and entropy Δ*S*_M_ of melting

Ionic liquid	*T*_C_/K	*T*_G_/K	*T*_M_/K	Δ*H*_M_/kJ mol^−1^	Δ*S*_M_/J K^−1^ mol^−1^
[P(3O1)_4_]Br	245.1 ± 1.1	—	325.7 ± 1.3	34.7 ± 1.8	106.5 ± 5.6
[P(3O1)_4_][NTf_2_]	—	190.9 ± 0.9	—	—	—
[P(3O1)_4_][B(CN)_4_]	—	193.0 ± 0.8	—	—	—
[P(3O1)_4_][BF_4_]	—	200.3 ± 1.0	279.8 ± 0.4	21.9 ± 0.5	78.2 ± 1.8
[P(3O1)_4_][PF_6_]	243.4 ± 3.8	—	324.6 ± 0.4	42.1 ± 0.4	129.7 ± 1.1
[P5555]Br	304.6 ± 0.4	—	336.8 ± 0.4	10.6 ± 0.6	31.5 ± 1.7
[P5555][NTf_2_]	294.7 ± 1.0	—	318.8 ± 0.2	14.8 ± 0.1	46.3 ± 0.1
[P5555][B(CN)_4_]	312.3 ± 1.0	—	382.8 ± 0.3	11.1 ± 0.2	29.1 ± 0.5
[P5555][BF_4_]	323.8 ± 1.9	—	358.5 ± 0.2	13.3 ± 0.1	37.2 ± 0.2
[P5555][PF_6_]	322.4 ± 1.5	—	344.3 ± 0.2	14.7 ± 1.1	42.7 ± 3.1

Most of the investigated ionic liquids crystallised at *T*_C_ upon cooling, and only three of the ether-containing ionic liquids solely formed glasses at *T*_G_. From these, [P(3O1)_4_][BF_4_] crystallised fully in the heating step (cold-crystallisation at *T*_CC_), so that the melting point *T*_M_ and the thermodynamic data for the fusion could be obtained. For the samples [P(3O1)_4_][NTf_2_] and [P(3O1)_4_][B(CN)_4_], only glass transitions could be obtained in various attempts. Together the samples cover all the three archetypical cooling and heating traces commonly found for ionic liquids.^32^ Besides [P(3O1)_4_]Br, all samples with the ether cation were obtained in the liquid state at room temperature. Although [P(3O1)_4_][PF_6_] has a melting point above room temperature, the dried sample remained liquid for months in a sealed flask. Therefore, the physicochemical properties of [P(3O1)_4_][PF_6_] could be measured easily in the supercooled state. Crystallisation of this sample was only observed upon strong supercooling in the DSC, and was even absent in some cases, forming solely a glass (followed by *T*_CC_).

All ionic liquids with the [P(3O1)_4_]^+^ cation show significantly lower melting points than their [P5555]^+^ counterparts. This is illustrated for the samples with the [BF_4_]^−^ anion, Fig. 2, where the lowering of the melting points leads to the formation of a room temperature ionic liquid with both symmetrical cation and anion. On average, the ether-substituted samples melt approximately 40 K lower than the alkyl-substituted samples. Furthermore, there are significant differences in the extent of supercooling. While the difference between crystallisation and melting is approximately 80 K for the [P(3O1)_4_]^+^ cation with bromide or [PF_6_]^−^ anion, the average difference is ≈37 K for the ionic liquids with the [P5555]^+^ cation.

The melting enthalpies of the ether substituted ionic liquids Δ*H*_M_ are significantly higher than for those with the [P5555]^+^ cations. The average melting enthalpy for the [P(3O1)_4_]^+^ ionic liquids is ≈33 kJ mol^−1^, more than 2.5 fold higher than the average of the [P5555]^+^ samples, with them being ≈13 kJ mol^−1^. The melting entropy Δ*S*_M_ is also significantly higher for the cations containing ether functionalities. While the [P5555]^+^ samples have an average Δ*S*_M_ of 37 J mol^−1^ K^−1^, the average of the ether substituted ionic liquids is with 105 J mol^−1^ K^−1^, approximately 2.8 times as high. For both enthalpy and entropy, the differences between non-functionalised and functionalised cations are highest for the bromide anion and lowest for [BF_4_]^−^. The obtained values are in good agreement with the values from Endo *et al.*, where an average Δ*H*_M_ of 20.2 kJ mol^−1^ and an average Δ*S*_M_ of 64.7 J mol^−1^ K^−1^ from a set of 257 different ionic liquids was reported.^13^ However, due to the high diversity of ionic liquids in general, there is also significant variation in the values for the enthalpy and entropy of fusion.

### Structure

**Fig. 3:**
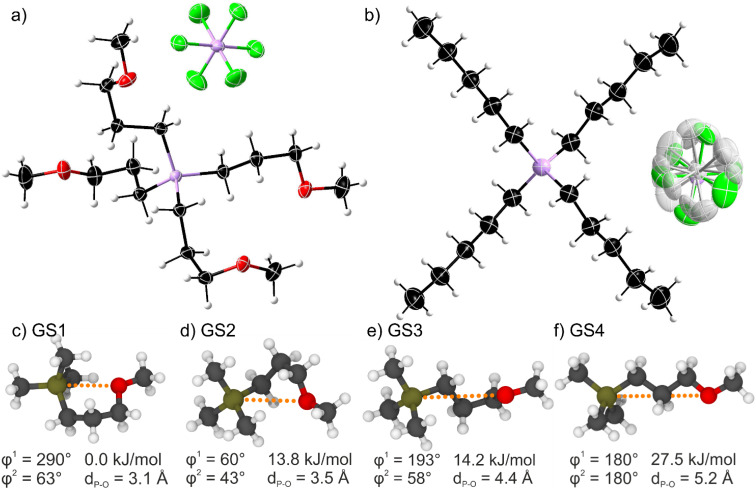
Top: Molecular structure of (a) the ether-functionalised [P(3O1)_4_][PF_6_] and (b) the non-functionalised [P5555][PF_6_] as obtained by X-ray diffraction. Symmetry transformations for the displayed [PF_6_]^−^ anion are 1 − *x*, 1 − *y*, 1 − *z* for (a) and 1/2 + *x*, 3/2 − *y*, 1/2 + *z* for (b). The shaded atoms represent the disordering with the lower side occupation factors. The disordered atoms with the lower side occupancy factors are represented by shaded colors. Color code: C black, O red, F green, P pink, H light grey. Bottom: (c)–(f) *ab initio* gas phase structures labelled GS1-4 showing the minimum energy conformers of a 3-methoxypropyl side chain (*cf.* ESI Fig. S11 and Table S13†).

Single crystal analysis allows insight into the conformation and arrangement of ions in bulk state. It is generally assumed that the energetic situation in the liquid state is similar to the crystal, only that the long-range spatial order is lost.^33^ The crystal structures of the two ionic liquids with [PF_6_]^−^ anions are shown in Fig. 3. The [P(3O1)_4_]^+^ cation shows a more compact structure with curled side chains. This structure is the result of the C_α_-C_β_-C_γ_-O torsion angle being ≈60° for the four inequivalent side chains (see ESI† for details), giving an average P_cation_⋯O distance of 4.47 Å. On the contrary, the alkyl-substituted cation has four equivalent linear side chains with a C_α_-C_β_-C_γ_-C_δ_ torsion angle close to 180°. The distance between the cation-phosphorus centre and the carbon in delta position is 5.35 Å, thus significantly larger than for the ether analogues.

We performed *ab initio* calculations in the gas phase on simple model cations such as the (3-methoxypropyl)trimethylphosphonium cation. Details including two-dimensional scans of the potential energy surface can be found in the ESI.† Critically, four relevant minimum energy structures were observed, Fig. 3c–f. Three curled structures are thermally accessible (within 6 kT ≈ 15 kJ mol^−1^ at room temperature). The energy of these structures increases with the P_cation_⋯O distance. Thus, curled structures are energetically favoured, and the linear conformation is too high to be significantly populated at room temperature (Fig. 3f).

The *ab initio* simulations from the model system were used to parameterise a force field for classical molecular dynamics simulations. Interestingly, the structures observed in the bulk liquid simulation of [P(3O1)_4_][BF_4_] correspond to GS3 and, to a lesser extent, GS2. For the alkyl substituted cation, *i.e*. in the bulk liquid simulation of [P5555][BF_4_], by far the most prevalent conformer is the linear one. These observations are consistent with the experimental crystal structures, in which the cations exclusively assume the conformer which is most populated in the simulations.

The trends in cation–anion distances differ between experimental crystal structures and the bulk liquid simulations. In the crystal, the cation–anion distance (P_cation_⋯P_anion_) is 5.11 Å for [P(3O1)_4_][PF_6_] and 5.60 Å for [P5555][PF_6_]. In contrast, for the bulk liquid simulation, cation–anion distances are larger for [P(3O1)_4_][BF_4_] compared to [P5555][BF_4_] (see ESI†). This complete reversal of trends matches the observed higher melting enthalpies of ionic liquids with ether functionalised cations (*cf.*Table 1). For scale, simply moving two unlike point charges of ±1*e* apart from 5.11 Å to 5.60 Å already comes with an energetic cost of approximately 24 kJ mol^−1^. Furthermore, the cations show little disorder in the crystal structures, again in line with the trends in melting enthalpy.

### Configurational entropy

**Fig. 4:**
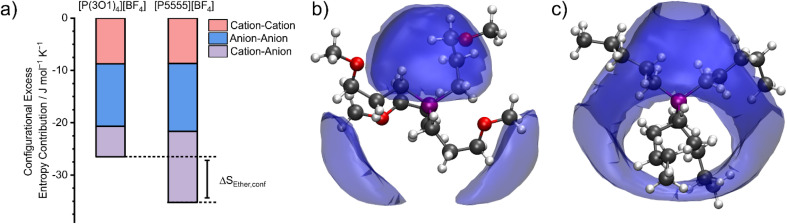
(a) Contributions to the configurational excess entropy obtained from the radial distribution function (angular disorder is not considered). Spatial distribution functions showing the coordination of anions around cations are shown in (b) for [P(3O1)_4_][BF_4_] and (c) for [P5555][BF_4_]. The isosurface is drawn at 10 mJ mol^−1^ K^−1^ Å^−3^.

The configurational entropy was calculated directly using the radial distribution functions from the molecular dynamics simulation.^34^ This straightforward approach has the advantage that cation–cation, cation–anion, and anion–anion contributions to the entropy can be separated. The results are shown in Fig. 4. Here, the entropy is given as excess entropy, *i.e.*, relative to the ideal gas. In contrast, Endo *et al.* reported the configurational contribution to the melting entropy, *i.e.* relative to the crystal, by subtracting other entropic contributions (conformation/translation/rotation/vibration) from Δ*S*_M_.^13,35,36^ The results are of similar magnitude but opposite sign.

Absolute accuracy is difficult to achieve with molecular dynamics simulations. Therefore, we will focus on the relative differences between the two simulations. Critically, the configurational excess entropy contribution of like-charge pairs are virtually identical for [P(3O1)_4_][BF_4_] and [P5555][BF_4_]. The main difference between the two simulations lies in the cation–anion configurational entropy. Here, the ether functionalisation leads to an increase in configurational entropy of approximately 8 J mol^−1^ K^−1^. The corresponding melting point depression Δ*T*_M_ can be estimated using eqn (2).2
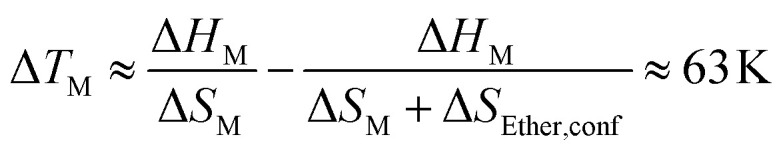
Here, Δ*H*_M_ and Δ*S*_M_ are the experimental enthalpy and entropy of melting for [P5555][BF_4_]. Thus, Δ*T*_M_ corresponds to the theoretical melting point depression after adding Δ*S*_Ether,conf_ (*cf.*Fig. 4). The seemingly small difference in the configurational entropies thus leads to a melting point depression of 63 K. While this is in good agreement with the experimentally observed melting point depression of 79 K, the experimentally observed difference in the entropy of melting is larger (41.0 J K^−1^ mol^−1^). The higher experimental entropy compared to the theoretical value can be explained with angular contributions which are not included in Fig. 4a. A more quantitative estimate can be obtained from the spatial distribution function (see ESI†). However, due to difficulties with convergence, we decided to choose the more robust approach based on the radial distribution functions.

The configurational excess entropy is directly related to the liquid structure. Pronounced maxima and minima in the radial distribution functions translate to more negative configurational excess entropy, *i.e.*, a loss of entropy compared to the ideal gas. The cation–anion spatial distribution functions in Fig. 4b/c qualitatively show the difference between ether- and alkyl functionalised cations. The isosurfaces are drawn at the same value for the excess entropy contribution per volume. Hence, the cation–anion structure is much less well defined for [P(3O1)_4_][BF_4_] compared to [P5555][BF_4_] due to the curling of the cations, which in turn leads to the difference in configurational entropies.§§Since the isosurfaces are drawn at the same threshold for the entropy, the isosurface which encompasses the larger volume corresponds to the more localised cation–anion structure, see also ESI. The radial distribution functions are visually identical, *cf.* ESI, eqn (11).

### Density and transport properties

**Fig. 5:**
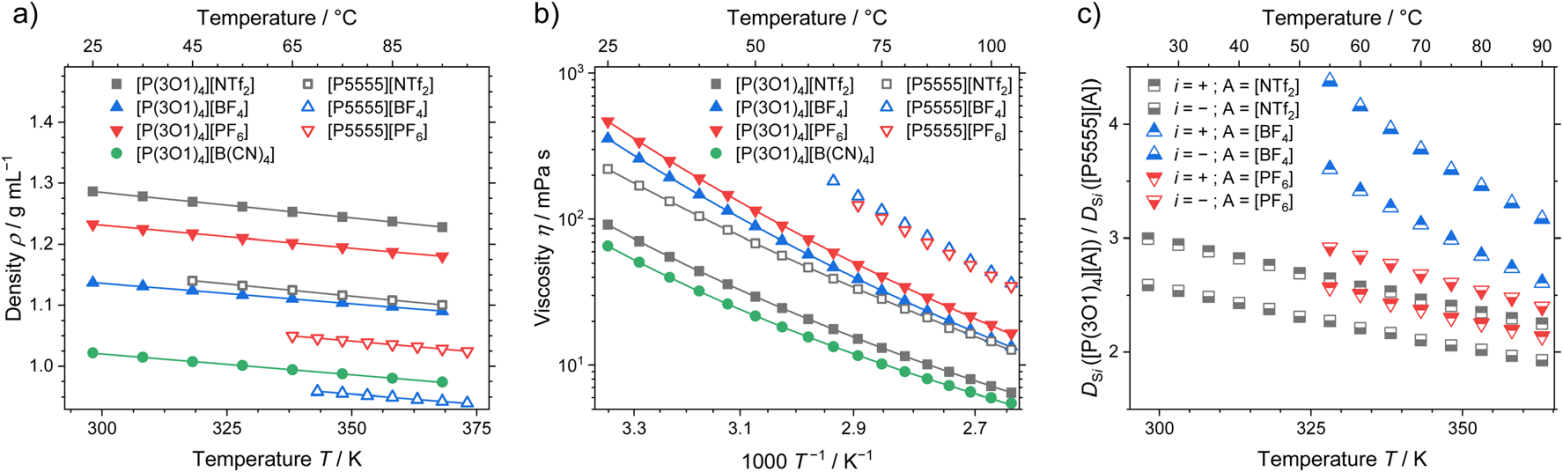
(a) Density, (b) viscosity and (c) ratio of the self-diffusion coefficients for the ether-functionalised and alkylated ionic liquids. Drawn lines are (a) the linear fit and (b) the fitting according to the Vogel–Fulcher–Tammann equation. Experimental values and fitting parameters are given in the ESI.†

Apart from the melting point depression, one of the key benefits of ether functionalisation is the significant acceleration of dynamics, which becomes clear when comparing the macroscopic properties of ionic liquids with the two cations. Density, viscosity, and ratio of the self-diffusion coefficients of ether functionalised to non-functionalised cations are shown in Fig. 5. The effects of the functionalisation on the other dynamic properties, conductivity and self-diffusion coefficients (see ESI† for experimental values and fitting), are similar to the viscosity and are therefore not discussed here.

The density of the ether containing ionic liquids is markedly higher than the ones with the [P5555]^+^ cation and the same anion, Fig. 5a. These systematically higher densities are the result of the more compact cation structures, as indicated from the crystal structure analysis and the MD simulations. With increasing anion weight, the difference in density also decreases. The largest difference in density is therefore found for the [BF_4_]^−^ anion, where the ionic liquid with the [P(3O1)_4_]^+^ cation has a 16% higher density, while for the [NTf_2_]^−^ anion only a 12% difference in density was found. In combination with the crystal structures, this partly contradicts the finding that the ether-functionalisation improves the dynamics of ionic liquids by increasing free volume due to highly flexible side chains.^31,37^

The viscosity as macroscopic quantity reveals significantly accelerated dynamics of the ionic liquids with the [P(3O1)_4_]^+^ cation, which are independent of the anion, Fig. 5b. The much lower viscosities of the ether ionic liquids persist over the whole investigated temperature range up to 378 K. Besides the comparably high melting points, the slow dynamics of ionic liquids, especially around room-temperature, are a major obstacle for technical implementations. Therefore, using the ether-substitution strategy to increase the configurational entropy also gives a powerful boost to the dynamics, which is beneficial for a broad range of applications.

The microscopic transport process of self-diffusion is complementary to the macroscopic dynamics. The ratio of the cation and anion self-diffusion coefficients of ether-substituted to non-functionalised ionic liquids are shown in Fig. 5c. Similar to the ratios of the viscosities, the ratios of the self-diffusion coefficients decrease with temperature, yet remain significant. The accelerated microscopic dynamics are thereby reflected by the faster dynamics of both cation and anion to a similar extent. This shows that the ether-functionalisation affects the dynamics of both ions, independent of the anion used.

## Conclusions

In this manuscript we investigated room temperature ionic liquids with small and highly symmetric ions. To achieve the desired low melting points, we used a phosphonium cation with four identical ether side chains, specifically [P(3O1)_4_]^+^. Ionic liquids with a structurally comparable cation with methylene instead of ether groups were included in the study for comparison.

The ionic liquids with ether functionalised cations all showed consistently and significantly lowered thermal transitions despite higher melting enthalpies and little cation disorder in the crystal structures. This somewhat counterintuitive result can be fully explained with a significant entropic contribution in all ionic liquids with the [P(3O1)_4_]^+^ cation. Molecular dynamics simulations revealed a significant difference in the configurational entropy between the two ionic liquids [P(3O1)_4_][BF_4_] and [P5555][BF_4_]. The largest contribution was due to more diffuse cation–anion arrangements in [P(3O1)_4_][BF_4_], and this contribution alone would be expected to lead to a melting point depression close to what is observed experimentally.

The origin of the enthalpic and entropic contributions can be understood on a molecular level by comparing the experimental crystal structures with the results from *ab initio* and molecular dynamics simulations. In the crystal structure, cation–anion distances are shorter for ether-functionalised cations and longer for alkyl-functionalised cations. In the molecular dynamics simulation of the bulk liquid, the exact opposite situation was observed. Thus, from a simple electrostatic viewpoint, ether functionalisation enthalpically stabilises the crystal and destabilises the liquid, leading to a higher melting enthalpy.

A single conformer, corresponding to GS3 of the *ab initio* model, is observed in the crystal structures involving the [P(3O1)_4_]^+^ cation. This conformer is also the most prevalent in the molecular dynamics simulations of the bulk liquid. Additionally, the GS2 conformer with more pronounced curling is observed in the liquid simulation (but not the crystal structures). In contrast, the side chains in the [P5555]^+^ cation predominantly assume linear conformations in all three states of matter. The curling of ether functionalised cations leads to a more diffuse cation–anion arrangement and exceptionally low excess (*i.e.* high configurational) entropy.

In addition to lowered thermal transitions, we observed fast dynamics and competitive transport properties of the symmetric ionic liquids. For example, the viscosity of the well-known ionic liquid 1-butyl-3-methylimidazolium bis(trifluoromethylsulfonyl)imide [BMIM][TFSI] at room temperature is around 51 mPa s.^38^ The viscosity of [P(3O1)_4_][B(CN)_4_], 66 mPa s at room temperature, was only marginally higher. From a practical perspective, two points are important to mention here.

Firstly, it is very likely that the viscosity can be further reduced by using [P(2O1)_4_]^+^ instead of [P(3O1)_4_]^+^. This shorter side chain is still capable of curling, and the reduced ion volume and mass can be expected to lower the viscosity. Additional *ab initio* simulations regarding the position of the oxygen atom in the side chain can be found in the ESI.† Recent work by Yoshii *et al.* revealed excellent performance even of (asymmetric) pyrrolidinium ionic liquids with only one spacer between quaternary centre and oxygen atom,^19^ suggesting that [P(1O1)_4_]^+^ might also be a promising candidate. However, issues with ylide formation and reduced stability of (2O1) and (1O1) functionalised cations can be anticipated.

Secondly, symmetric ions with identical side chains are more straightforward and economical to synthesise than their asymmetric counterparts. In this work, we chose the Grignard route for reasons of safety and reproducibility; this route is not possible for ethylene spacers due to β-oxygen elimination. Alternative synthetic routes would require the use of PH_3_, which we chose to avoid.

In summary, we present novel ionic liquids with low melting points and fast dynamics despite being composed from two symmetric ions. The importance of configurational entropy in explaining such behaviour is evident, in line with previous work from the community. These unparalleled results open new pathways for the design of ionic liquids and provide guidance for the next steps in our efforts to understand the physics of ionic liquids.

## Data availability

The relevant data is contained in the ESI.†

## Author contributions

D. R.: conceptualisation, formal analysis, investigation (syntheses, physicochemical measurements), validation, project administration, visualisation, writing – original draft, writing – review & editing. F. P.: conceptualisation, data curation, formal analysis, funding acquisition, investigation (syntheses, theoretical chemistry), methodology, project administration, software, visualisation, writing – original draft, writing – review & editing. D. S.: investigation (syntheses, physicochemical measurements), writing – original draft, writing – review & editing. B. M.: data curation, investigation (crystallography), writing – review & editing. A. W.: data curation, investigation (crystallography), writing – review & editing. M. J.: data curation, investigation (rheology), writing – review & editing. T. W.: funding acquisition, project administration, supervision, writing – review & editing. C. K.: funding acquisition, project administration, supervision, writing – review & editing.

## Conflicts of interest

There are no conflicts to declare.

## Supplementary Material

SC-014-D3SC03240J-s001

SC-014-D3SC03240J-s002

SC-014-D3SC03240J-s003
