# Supplementary material for: Room temperature ionic liquids with two symmetric ions
Source: Chem Sci. 2023 Sep 8;14(37):10340–6. doi: 10.1039/d3sc03240j (PMC10530934; doi:10.1039/d3sc03240j)
Supplement: SC-014-D3SC03240J-s002 [file SC-014-D3SC03240J-s002.pdf]

Electronic Supplementary Material (ESI) for Chemical Science.
This journal is © The Royal Society of Chemistry 2023

Supporting Information to:

Room Temperature Ionic Liquids with Two Symmetric Ions

Daniel Rauber[1*†], Frederik Philippi[2*†], Daniel Schroeder[1], Bernd Morgenstern[1], Andrew J. P. White[2], Marlon Jochum[3], Tom Welton[2], Christopher W.M. Kay[1,4*]

[1] Department of Chemistry, Saarland University, Campus B 2.2, 66123 Saarbrücken, Germany. E-mail: daniel.rauber@uni-saarland.de

[2] Department of Chemistry, Molecular Sciences Research Hub, Imperial College London, White City Campus, London W12 0BZ, UK. E-mail: f.philippi18@imperial.ac.uk

[3] INM-Leibniz Institute for New Materials, Campus D2.2, 66123 Saarbrücken, Germany.

[4] London Centre for Nanotechnology, University College London, 17-19 Gordon Street, London WC1H 0AH, UK. E-mail: c.kay@ucl.ac.uk

[†] These authors contributed equally to the manuscript.

Contents

1. Preparation of the Investigated Ionic Liquids 3
1.1 Materials 3
1.2 Preparation of the phosphine precursors 3
1.2.1 Tris(3-methoxypropyl)phosphine 3
1.2.2 Tripentylphosphine 4
1.3 Preparation of the Ionic liquids 4
1.3.1 Tetrakis(3-methoxypropyl)phosphonium bromide $[P(3O1)_4]Br$ 4
1.3.2 Tetrakis(3-methoxypropyl)phosphonium bis(trifluoromethanesulfonyl)imide $[P(3O1)_4][NTf_2]$ ... 5
1.3.3 Tetrakis(3-methoxypropyl)phosphonium tetracyanoborate $[P(3O1)_4][B(CN)_4]$ 5
1.3.4 Tetrakis(3-methoxypropyl)phosphonium tetrafluoroborate $[P(3O1)_4][BF_4]$ 6
1.3.5 Tetrakis(3-methoxypropyl)phosphonium hexafluorophosphate $[P(3O1)_4][PF_6]$ 6
1.3.6 Tetrapentylphosphonium bromide [P5555]Br 6
1.3.7 Tetrapentylphosphonium bis(trifluoromethanesulfonyl)imide $[P5555][NTf_2]$ 7
1.3.8 Tetrapentylphosphonium tetracyanoborate $[P5555][B(CN)_4]$ 7
1.3.9 Tetrapentylphosphonium tetrafluoroborate $[P5555][BF_4]$ 8
1.3.10 Tetrapentylphosphonium hexafluorophosphate $[P5555][PF_6]$ 8
2. Physical Properties of the Ionic Liquids 9
2.1 Differential Scanning Calorimetry (DSC) 9
2.2 Density 12
2.3 Viscosity 13
2.4 Specific Conductivity 15
2.5 Molar Conductivity 17
2.6 Self-diffusion Coefficients 19
3. Ab initio simulations 23
4. MD simulations 27
4.1 Force field development 27
4.2 Preparation and equilibration 28
4.3 Trajectory analyses 28
5. Crystal Structure Analysis 38
5.1 Refinement details 38
5.2 Crystal Data and Structure Refinement 39
6. References 45

1. Preparation of the Investigated Ionic Liquids

The absence of halide impurities was checked by testing the ionic liquids with 0.1 M aqueous silver nitrate solution. Prior to all physicochemical measurements, the samples were dried in high vacuum while stirring for at least two days. In this work we used a two-step approach for the synthesis of the phosphonium halides: first, preparation of the phosphine *via* Grignard/PCl_3; second, alkylation with an organic halide. In practice, the symmetry of the cation can be exploited to synthesize the cation precursors in one step, for example by direct alkylation of PH_3 (or NH_3 for ammonium ionic liquids).[1]

1.1 Materials

Phosphorus trichloride (99%) from Sigma Aldrich (USA) was distilled prior to use and stored under argon. 1-bromo-3-methoxypropane (>98%) from TCI Europe (Belgium) was dried with 4Å molecular sieves, filtered, distilled under argon and stored over 4Å molecular sieves and under argon. Lithium bis(trifluoromethanesulfonyl)imide (99%), sodium tetrafluoroborate (>98%) as well as potassium hexafluroborate (99%) were obtained from IoLiTec (Germany) and used as received. Potassium tetracyanoborate was synthesized as reported previously.[2] Nuclear magnetic resonance (NMR) spectra were recorded on an AVANCE II 400 NMR spectrometer (Bruker, USA) using the residual solvent signal as reference. The preparation of phosphine precursors as well as the corresponding alkylation reactions were performed under Schlenk conditions using Argon as inter gas.

1.2 Preparation of the phosphine precursors

Details on the phosphine synthesis, such as the choice of solvents, can be found in https://doi.org/10.25560/98938.

1.2.1 Preparation of Tris(3-methoxypropyl)phosphine

16.1 g fresh magnesium turnings (661 mmol / 3.22 eq) were placed in a 1000 mL three necked round bottom flask equipped with overhead stirrer and activated by dry stirring in vacuum. A total of 75.5 mL 1-bromo-3-methoxypropane (102 g / 663 mmol / 3.23 eq) dissolved in 350 mL dry THF were added dropwise over the course of 3 h. After addition, the reaction mixture was stirred for 3 h and then cooled to −78°C (isopropanol / dry ice bath). 18 mL phosphorus trichloride (28.1 g / 205 mmol / 1.00 eq) dissolved in 75 mL dry diethyl ether were added dropwise over 25 min. After completion of the addition, the reaction was allowed to warm to room temperature and stirred for 17 h. The reaction mixture was then quenched by slow addition of 100 mL half saturated ammonium chloride solution. The organic solvent was removed *via* cannula, and the residue extracted with 100 mL diethyl ether. The ethereal phases were combined and the organic solvent removed by means of reduced pressure. The residual containing the raw phosphine was distilled twice in fine vacuum (93°C to 94°C, pressure between 3.8×10^{-2} mbar and 6.0×10^{-2} mbar). Thus, 30.2 g tris(3-methoxypropyl)phosphine (120 mmol / 59% isolated yield) were obtained. The purity was 95% as determined by ^{31}P NMR.

^{1}H-NMR (400 MHz, $CDCl_3$): δ/ppm = 3.37 (t, $^3J_{HH}$ = 6.5 Hz, 2H, ***CH***$_2$-O-CH_3), 3.30 (s, 3H, -CH_2-O-***CH***$_3$), 1.73-1.58 (m, 2H, P-CH_2-***CH***$_2$), 1.48-1.32 (m, 2H, P-***CH***$_2$-CH_2).

^{13}C{^{1}H}-NMR (101 MHz, $CDCl_3$): δ/ppm = 74.09 (d, $^3J_{CP}$ = 11.9 Hz, -***CH***$_2$-O-CH_3), 58.59 (s, -CH_2-O-***CH***$_3$), 26.78 (d, $^2J_{CP}$ = 13.2 Hz, P-CH_2-***CH***$_2$), 23.96 (d, $^1J_{CP}$ = 12.6 Hz, P-***CH***$_2$).

^{31}P{^{1}H}-NMR (162 MHz, $CDCl_3$): δ/ppm = −30.41.

1.2.2 Preparation of Tripentylphosphine

Following the literature protocol,[3] the Grignard reagent was prepared under Schlenk conditions. To this end, 15.0 g Magnesium turnings (617 mmol / 3.09 eq) were activated by dry stirring under vacuum, and 3 mL bromopentane (3.55 g / 23.4 mmol / 0.12 eq) in 50 mL dry diethyl ether were added. After the initial exothermic reaction ceased, 77 mL bromopentane (92.8 g / 612 mmol / 3.06 eq) in 300 mL dry diethyl ether were added dropwise over 5 h, followed by heating to reflux for 2 h. Then, the reaction mixture was cooled in a dry ice / ethanol bath, and 27.5 g phosphorus pentachloride (200 mmol / 1.00 eq) in 60 mL dry diethyl ether were added dropwise over the course of 2 h. After addition was complete, the reaction mixture was stirred at ambient temperature for 15 h, and quenched by addition of 60 mL half saturated aqueous ammonium chloride solution. The organic phase was decanted, and the diethyl ether distilled off. Vacuum distillation of the residue gave 29.1 g of raw product, which contained undesired phosphorus species and was further purified by vacuum distillation from potassium hydroxide, yielding 28.9 g tripentyl phosphine (118 mmol / 59% isolated yield).

^{1}H-NMR (400 MHz, $CDCl_3$): δ/ppm = 1.60-1.15 (m, 24H, P-($\boldsymbol{CH_2}$)$_4$-CH_3), 0.89 (t, $^3J_{HH}$ = 6.7 Hz, 9H, P-$(CH_2)_4$-$\boldsymbol{CH_3}$).

^{13}C{^{1}H}-NMR (101 MHz, $CDCl_3$): δ/ppm = 33.84 (d, $^3J_{CP}$ = 10.4 Hz, P-$(CH_2)_2$-$\boldsymbol{CH_2}$), 27.31 (d, $^2J_{CP}$ = 11.6 Hz, P-CH_2-$\boldsymbol{CH_2}$), 25.72 (d, $^1J_{CP}$ = 12.3 Hz, P-$\boldsymbol{CH_2}$), 22.53 (s, P-$(CH_2)_3$-$\boldsymbol{CH_2}$), 14.10 (s, $\boldsymbol{CH_3}$)

^{31}P{^{1}H}-NMR (162 MHz, $CDCl_3$): δ/ppm = −30.72 (s).

1.3 Preparation of the Ionic liquids

1.3.1 Preparation of Tetrakis(3-methoxypropyl)phosphonium bromide [P(3O1)$_4$]Br

The quaternary phosphonium compound was synthesized from 23.5 g tris(3-methoxypropyl)-phosphine (93.9 mmol / 1.0 eq.) and 13.7 mL of 1-bromo-3-methoxypropan (122 mmol / 18.7 g / 1.3 eq.) dissolved in mL dry, degassed acetonitrile under argon. The mixture was stirred for seven days at 35°C. After the completion of the reaction the solvent and excess reagent were removed by rotary evaporation. The residue was dried on a Schlenk line for two days giving the product in 98% yield (92.0 mmol / 37.1 g) as a colorless solid.

^{1}H-NMR (400 MHz, $CDCl_3$): δ/ppm = 3.45 (t, $^3J_{HH}$ = 5.6 Hz, 8H, CH_2-O), 3.30 (s, 12H, CH_3), 2.59 – 2.47 (m, 8H, P-CH_2), 1.93 – 1.79 (m, 8H, P-CH_2-$\mathbf{CH_2}$).

^{13}C{^{1}H}-NMR (101 MHz, $CDCl_3$): δ/ppm = 71.36 (d, $^3J_{CP}$ = 13.6 Hz, CH_2-O), 58.83 (s, CH_3), 22.44 (d, $^2J_{CP}$ = 4.4 Hz, P-CH_2-$\mathbf{CH_2}$), 17.02 (d, $^1J_{CP}$ = 49.6 Hz, P-CH_2).

^{31}P{^{1}H}-NMR (162 MHz, $CDCl_3$): δ/ppm = 36.00 (s).

1.3.2 Preparation of Tetrakis(3-methoxypropyl)phosphonium bis(trifluoromethanesulfonyl)-imide $[P(3O1)_4][NTf_2]$

The title compound was synthesized from 6.00 g of tetrakis(3-methoxypropyl)phosphonium bromide (14.9 mmol / 1.0 eq.) and 5.12 g lithium bis(trifluoromethanesulfonyl)imide (17.9 mmol / 1.2 eq.) in a biphasic mixture of approximately 20 mL of deionized water and 200 mL of dichloromethane. The mixture was stirred for 16 hours before the organic phase was separated and washed several times with about 20 mL of deionized water. The final washings were tested for the absence of halides with aqueous silver nitrate solution. After negative test for bromide, the organic solvent was removed on a rotary evaporator and dried in high vacuum at 50°C with stirring for two days. The title compound was obtained in 97% yield (14.4 mmol / 8.71 g) as a colorless liquid.

^{1}H-NMR (400 MHz, $CDCl_3$): δ/ppm = 3.41 (t, $^{3}J_{HH}$ = 5.5 Hz, 8H, CH_2-O), 3.29 (s, 12H, CH_3), 2.28 – 2.16 (m, 8H, P-CH_2), 1.86 – 1.72 (m, 8H, P-CH_2-**CH_2**).

^{13}C{^{1}H}-NMR (101 MHz, $CDCl_3$): δ/ppm = 119.95 (q, $^{1}J_{CF}$ = 321.5 Hz, CF_3), 71.06 (d, $^{3}J_{CP}$ = 13.0 Hz, CH_2-O), 58.65 (s, CH_3), 21.99 (d, $^{2}J_{CP}$ = 4.4 Hz, P-CH_2-**CH_2**), 16.49 (d, $^{1}J_{CP}$ = 49.9 Hz, P-CH_2).

^{19}F{^{1}H}-NMR (377 MHz, $CDCl_3$): δ/ppm = –78.89 (s)

^{31}P{^{1}H}-NMR (162 MHz, $CDCl_3$): δ/ppm = 36.52 (s).

1.3.3 Preparation of Tetrakis(3-methoxypropyl)phosphonium tetracyanoborate $[P(3O1)_4][B(CN)_4]$

The title compound was synthesized from 6.00 g of tetrakis(3-methoxypropyl)phosphonium bromide (14.9 mmol / 1.0 eq.) and 2.75 g potassium tetracyanoborate (17.9 mmol / 1.2 eq.) similar to $[P(3O1)_4][NTf_2]$. The title compound was obtained in 96% yield (14.3 mmol / 6.26 g) as a colorless liquid.

^{1}H-NMR (400 MHz, $CDCl_3$): δ/ppm = 3.47 (t, $^{3}J_{HH}$ = 5.4 Hz, 8H, CH_2-O), 3.35 (s, 12H, CH_3), 2.36 – 2.14 (m, 8H, P-CH_2), 1.92 – 1.78 (m, 8H, P-CH_2-**CH_2**).

^{11}B-NMR (128 MHz, $CDCl_3$): δ/ppm = –38.18 (s).

^{13}C{^{1}H}-NMR (101 MHz, $CDCl_3$): δ/ppm = 122.83 (q, $^{1}J_{CB}$ = 71.3 Hz, CN), 71.00 (d, $^{3}J_{CP}$ = 12.2 Hz, CH_2-O), 58.92 (s, CH_3), 22.25 (d, $^{2}J_{CP}$ = 4.5 Hz, P-CH_2-**CH_2**), 16.97 (d, $^{1}J_{CP}$ = 49.9 Hz, P-CH_2).

^{31}P{^{1}H}-NMR (162 MHz, $CDCl_3$): δ/ppm = 37.03 (s).

1.3.4 Preparation of Tetrakis(3-methoxypropyl)phosphonium tetrafluoroborate $[P(3O1)_4][BF_4]$

The title compound was synthesized from 7.00 g of tetrakis(3-methoxypropyl)phosphonium bromide (17.4 mmol / 1.0 eq.) and 2.29 g sodium tetrafluoroborate (20.8 mmol / 1.2 eq.) similar to $[P(3O1)_4][NTf_2]$. The title compound was obtained in 96% yield (16.8 mmol / 6.91 g) as a colorless liquid.

^{1}H-NMR (400 MHz, $CDCl_3$): δ/ppm = 3.43 (t, $^{3}J_{HH}$ = 5.5 Hz, 8H, CH_2-O), 3.29 (s, 12H, CH_3), 2.34 – 2.22 (m, 8H, P-CH_2), 1.87 – 1.75 (m, 8H, P-CH_2-**CH_2**).

^{11}B-NMR (128 MHz, $CDCl_3$): δ/ppm = –1.06 (s).

^{13}C{^{1}H}-NMR (101 MHz, $CDCl_3$): δ/ppm = 71.29 (d, $^{3}J_{CP}$ = 13.6 Hz, CH_2-O), 58.71 (s, CH_3), 22.05 (d, $^{2}J_{CP}$ = 4.4 Hz, P-CH_2-**CH_2**), 16.43 (d, $^{1}J_{CP}$ = 49.9 Hz, P-CH_2).

^{19}F{^{1}H}-NMR (377 MHz, $CDCl_3$): δ/ppm = –151.63 (s, $^{10}BF_4$), –151.68 (s, $^{10}BF_4$)

^{31}P{^{1}H}-NMR (162 MHz, $CDCl_3$): δ/ppm = 36.21 (s).

1.3.5 Preparation of Tetrakis(3-methoxypropyl)phosphonium hexafluorophosphate $[P(3O1)_4][PF_6]$

The title compound was synthesized from 6.00 g of tetrakis(3-methoxypropyl)phosphonium bromide (14.9 mmol / 1.0 eq.) and 3.29 g potassium hexafluorophosphate (17.9 mmol / 1.2 eq.) similar to $[P(3O1)_4][NTf_2]$. The title compound was obtained in 98% yield (14.6 mmol / 6.83 g) as a colorless liquid.

^{1}H-NMR (400 MHz, $CDCl_3$): δ/ppm = 3.39 (t, $^{3}J_{HH}$ = 5.9 Hz, 8H, CH_2-O), 3.26 (s, 12H, CH_3), 2.33 – 2.19 (m, 8H, P-CH_2), 1.81 – 1.67 (m, 8H, P-CH_2-**CH_2**).

^{13}C{^{1}H}-NMR (101 MHz, $CDCl_3$): δ/ppm = 71.21 (d, $^{3}J_{CP}$ = 15.6 Hz, CH_2-O), 57.90 (s, CH_3), 21.03 (d, $^{2}J_{CP}$ = 4.0 Hz, P-CH_2-**CH_2**), 14.91 (d, $^{1}J_{CP}$ = 49.5 Hz, P-CH_2).

^{19}F{^{1}H}-NMR (377 MHz, $CDCl_3$): δ/ppm = –70.17 (d, $^{1}J_{FP}$ = 711.4 Hz)

^{31}P{^{1}H}-NMR (162 MHz, $CDCl_3$): δ/ppm = 36.61 (s, P-CH_2), –144.20 (hept, $^{1}J_{PF}$ = 711.5 Hz, PF_6).

1.3.6 Preparation of Tetrapentylphosphonium bromide [P5555]Br

The title compound was synthesized from 25.0 mL of tripentylphosphine (20.3 g / 83.1 mmol / 1.0 eq.) and 14.9 mL pentyl bromide (18.2 g / 120 mmol / 1.45 eq.) in 300 mL of dry, degassed acetonitrile under argon atmosphere. After stirring at ambient temperature for three days the solvent and excess bromo alkane were removed by rotary evaporation. The residue was dried for two days in oil pump vacuum to obtain title compound in 99% yield (82.3 mmol / 32.5 g) as a colorless solid.

^{1}H-NMR (400 MHz, $CDCl_3$): δ/ppm = 2.50 – 2.39 (m, 8H, P-CH_2), 1.74 – 1.41 (m, 16H, P-CH_2-(**CH_2**)$_2$), 1.41 – 1.19 (m, 8H, **CH_2**-CH_3), 0.90 (t, $^{3}J_{HH}$ = 7.2 Hz, 12H, CH_3).

^{13}C{^{1}H}-NMR (101 MHz, $CDCl_3$): δ/ppm = 32.93 (d, $^{3}J_{CP}$ = 14.6 Hz. P-(CH_2)$_2$-**CH_2**), 22.15 (s, **CH_2**-CH_3), 21.74 (d, $^{2}J_{CP}$ = 4.8 Hz, P-CH_2-**CH_2**), 19.50 (d, $^{1}J_{CP}$ = 47.0 Hz, P-CH_2), 13.84 (s, CH_3).

^{31}P{^{1}H}-NMR (162 MHz, $CDCl_3$): δ/ppm = 32.69 (s).

1.3.7 Preparation of Tetrapentylphosphonium bis(trifluoromethanesulfonyl)imide [P5555][NTf_2]

[P5555][NTf_2] was prepared in a biphasic water/dichloromethane mixture similar to [P(3O1)$_4$][NTf_2] using 7.00 g tetrapentylphosphonium bromide (17.7 mmol / 1.0 eq.) in combination with 6.10 g lithium bis(trifluoromethanesulfonyl)imide (21.2 mmol / 1.2 eq.). After drying in high vacuum for two days, the ionic liquid was obtained in 99% yield (21.0 mmol / 12.5 g) as a colorless solid.

^{1}H-NMR (400 MHz, $CDCl_3$): δ/ppm = 2.43 – 1.94 (m, 8H, P-CH_2), 1.68 – 1.41 (m, 16H, P-CH_2-(**CH_2**)$_2$), 1.41 – 1.23 (m, 8H, **CH_2**-CH_3), 0.91 (t, $^{3}J_{HH}$ = 7.2 Hz, 12H, CH_3).

^{13}C{^{1}H}-NMR (101 MHz, $CDCl_3$): δ/ppm = 120.04 (q, $^{1}J_{CF}$ = 321.3 Hz, CF_3), 32.73 (d, $^{3}J_{CP}$ = 14.6 Hz. P-(CH_2)$_2$-**CH_2**), 21.98 (s, **CH_2**-CH_3), 21.36 (d, $^{2}J_{CP}$ = 4.7 Hz, P-CH_2-**CH_2**), 18.83 (d, $^{1}J_{CP}$ = 47.3 Hz, P-CH_2), 13.75 (s, CH_3).

^{19}F{^{1}H}-NMR (377 MHz, $CDCl_3$): δ/ppm = –78.81 (s)

^{31}P{^{1}H}-NMR (162 MHz, $CDCl_3$): δ/ppm = 32.98 (s).

1.3.8 Preparation of Tetrapentylphosphonium tetracyanoborate [P5555][B(CN)$_4$]

The tetracyanoborate salt was prepared similar to [P(3O1)$_4$][B(CN)$_4$] from 7.00 g tetrapentyl-phosphonium bromide (17.7 mmol / 1.0 eq.) in combination with 3.27 g potassium tetracyanoborate (21.2 mmol / 1.2 eq.). The product [P5555][B(CN)$_4$] was obtained in 98% yield (17.3 mmol / 7.47 g) as a colorless solid.

^{1}H-NMR (400 MHz, $CDCl_3$): δ/ppm = 2.13 – 2.01 (m, 8H, P-CH_2), 1.64 – 1.44 (m, 16H, P-CH_2-(**CH_2**)$_2$), 1.45 – 1.30 (m, 8H, **CH_2**-CH_3), 0.94 (t, $^{3}J_{HH}$ = 7.2 Hz, 12H, CH_3).

^{11}B-NMR (128 MHz, $CDCl_3$): δ/ppm = –38.20 (s)

^{13}C{^{1}H}-NMR (101 MHz, $CDCl_3$): δ/ppm = 122.75 (q, $^{1}J_{CB}$ = 71.3 Hz, CN), 32.72 (d, $^{3}J_{CP}$ = 14.4 Hz. P-(CH_2)$_2$-**CH_2**), 21.94 (s, **CH_2**-CH_3), 21.25 (d, $^{2}J_{CP}$ = 4.7 Hz, P-CH_2-**CH_2**), 18.88 (d, $^{1}J_{CP}$ = 47.2 Hz, P-CH_2), 13.73 (s, CH_3).

^{31}P{^{1}H}-NMR (162 MHz, $CDCl_3$): δ/ppm = 33.33 (s).

1.3.9 Preparation of Tetrapentylphosphonium tetrafluoroborate [P5555][BF_4]

The title ionic liquid was prepared similar to the other ionic liquids using 7.00 g of tetrapentyl-phosphonium bromide (17.7 mmol / 1.0 eq.) and 2.33 g sodium tetrafluoroborate (21.2 mmol / 1.2 eq.). The product was obtained in 98% yield (17.3 mmol / 6.98 g) as a colorless solid.

^{1}H-NMR (400 MHz, $CDCl_3$): δ/ppm = 2.31 – 2.19 (m, 8H, P-CH_2), 1.65 – 1.42 (m, 16H, P-CH_2-(**CH_2**)$_2$), 1.42 – 1.28 (m, 8H, **CH_2**-CH_3), 0.90 (t, $^{3}J_{HH}$ = 7.2 Hz, 12H, CH_3).

^{11}B-NMR (128 MHz, $CDCl_3$): δ/ppm = –1.08 (s).

^{13}C{^{1}H}-NMR (101 MHz, $CDCl_3$): δ/ppm = 32.89 (d, $^{3}J_{CP}$ = 14.6 Hz. P-(CH_2)$_2$-**CH_2**), 22.09 (s, **CH_2**-CH_3), 21.56 (d, $^{2}J_{CP}$ = 4.7 Hz, P-CH_2-**CH_2**), 19.02 (d, $^{1}J_{CP}$ = 47.2 Hz, P-CH_2), 13.82 (s, CH_3).

^{19}F{^{1}H}-NMR (377 MHz, $CDCl_3$): δ/ppm = –151.42 (s, $^{10}BF_4$), –151.48 (s, $^{10}BF_4$)

^{31}P{^{1}H}-NMR (162 MHz, $CDCl_3$): δ/ppm = 31.81 (s).

1.3.10 Preparation of Tetrapentylphosphonium hexafluorophosphate [P5555][PF_6]

The hexafluorophosphate ionic liquids was synthesized similar to [P(3O1)$_4$][PF_6] from 7.00 g of tetra-pentylphosphonium bromide (17.7 mmol / 1.0 eq.) and 3.90 g potassium hexafluorophosphate (21.2 mmol / 1.2 eq.). The title ionic liquid was obtained in 99% yield as a colorless solid (17.5 mmol / 8.07 g).

^{1}H-NMR (400 MHz, $CDCl_3$): δ/ppm = 2.17 – 2.05 (m, 8H, P-CH_2), 1.62 – 1.42 (m, 16H, P-CH_2-(**CH_2**)$_2$), 1.42 – 1.22 (m, 8H, **CH_2**-CH_3), 0.91 (t, $^{3}J_{HH}$ = 7.2 Hz, 12H, CH_3).

^{13}C{^{1}H}-NMR (101 MHz, $CDCl_3$): δ/ppm = 32.80 (d, $^{3}J_{CP}$ = 14.6 Hz. P-(CH_2)$_2$-**CH_2**), 22.01 (s, **CH_2**-CH_3), 21.37 (d, $^{2}J_{CP}$ = 4.8 Hz, P-CH_2-**CH_2**), 18.75 (d, $^{1}J_{CP}$ = 47.4 Hz, P-CH_2), 13.80 (s, CH_3).

^{19}F{^{1}H}-NMR (377 MHz, $CDCl_3$): δ/ppm = –72.17 (d, $^{1}J_{FP}$ = 712.8 Hz)

^{31}P{^{1}H}-NMR (162 MHz, $CDCl_3$): δ/ppm = 32.85 (s, P-CH_2), –144.20 (hept, $^{1}J_{PF}$ = 714.0 Hz, PF_6).

2. Physical Properties of the Ionic Liquids

2.1 Differential Scanning Calorimetry (DSC)

Thermal transitions were determined on a DSC 1 differential scanning calorimeter (Mettler Toledo Germany) equipped with liquid nitrogen. Samples were prepared by weighing approximately 10 mg of the ionic liquids in aluminum crucibles inside a Labmaster 130 glove box (MBraun, Germany). The hermetically sealed crucibles were pinned immediately before the closing of the DSC, where they were measured under inert gas flow. The temperature program started by heating from 298.15 K to 398.15K to remove the thermal history, followed by cooling to 153.15 K (cooling trace) and subsequently reheating to 398.15 K (heating trace). Different scanning rates and repeated measurements with various samples were used for the determination of the temperature T_X and enthalpy H_X of the thermal transitions. Temperatures for crystallization T_C, cold-crystallization T_{CC}, solid-solid transitions T_{SS} and melting point T_M are reported as the maximum of the enthalpic peaks. Glass transitions T_G were determined by the midpoint method. Temperatures of the thermal transitions and the corresponding enthalpies as obtained by differential scanning calorimetry (DSC) are summarized in Table S1, the resulting traces are shown in Figure S1.

The T-dependent transport properties Y were fitted by the empirical Vogel-Fulcher-Tammann (VFT) equation (S1)

$$Y = Y_0 \cdot \exp\left(\frac{B_Y}{T - T_{0,Y}}\right) \tag{S1}$$

With the fitted transport quantity Y (either η, κ, Λ_M or D_{Si}), and the material-dependent, empirical parameters Y_0, B_Y, and $T_{0,Y}$. For the viscosity B_η values are positive, as the viscosity decreases with temperature, will for the conductivities and the self-diffusion coefficients negative values of B_Y are found.

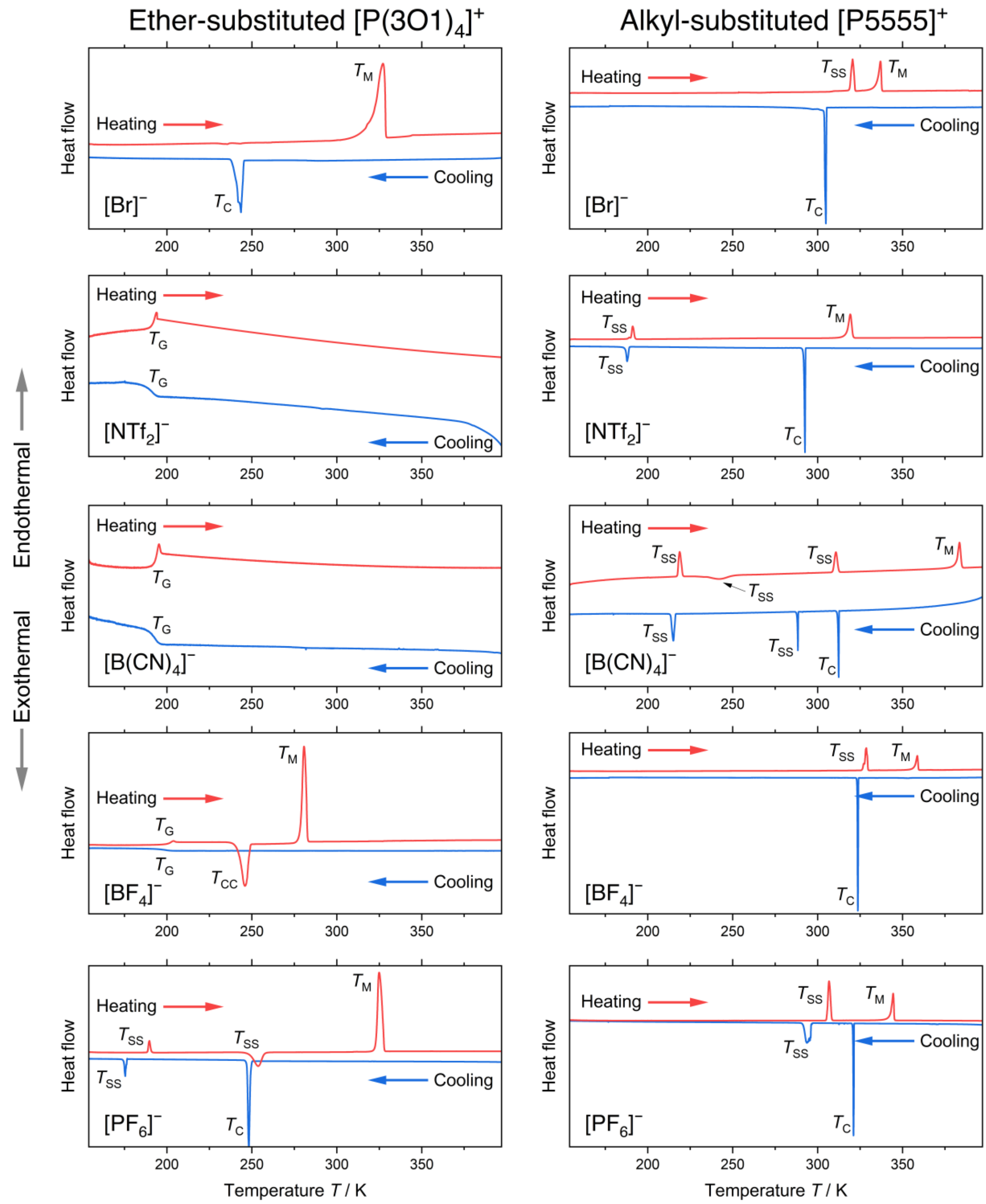

Figure S1: Cooling and heating traces of the investigated ionic liquids obtained by DSC. The obtained thermal transitions points are indicated in the graphs (T_C: crystallization, T_G: glass transition, T_M: melting; T_{CC}: cold-crystallization; T_{SS}: solid-solid transition).

Table S1: Results of the differential scanning calorimetry measurements with temperatures $\boldsymbol{T}$ and enthalpy $\Delta\boldsymbol{H}$ of the thermal transition (**C**: Crystallization (upon cooling); **CC**: Cold-crystallization (upon heating); **G**: Glass transition; **SS**: Solid-solid transitions; **M**: Melting). Endothermal events have a positive sign, exothermal events have a negative sign.

Ionic liquid	T_{C} / °C	ΔH_{C} / kJ mol^{-1}	T_{CC} / °C	ΔH_{CC} / kJ mol^{-1}	T_{G} / °C	T_{SS} / °C	ΔH_{SS} / kJ mol^{-1}	T_{M} / °C	ΔH_{M} / kJ mol^{-1}
[P(3O1)$_4$]Br	–28.1 ± 1.1	–25.4 ± 0.1	–	–	–	–	–	52.6 ± 1.3	34.7 ± 1.8
[P(3O1)$_4$][NTf$_2$]	–	–	–	–	–82.3 ± 0.9	–	–	–	–
[P(3O1)$_4$][B(CN)$_4$]	–	–	–	–	–80.2 ± 0.8	–	–	–	–
[P(3O1)$_4$][BF$_4$]	–	–	–33.1 ± 4.9	–16.8 ± 0.4	–72.9 ± 1.0	–	–	6.6 ± 0.4	21.9 ± 0.5
[P(3O1)$_4$][PF$_6$]	–29.8 ± 3.8	–19.0 ± 1.4	–	–	–	–97.7 ± 0.9 (upon cooling);<br>–84.1 ± 0.5 (upon heating)	–3.12 ± 0.01 (upon cooling);<br>2.70 ± 0.12 (upon heating)	51.4 ± 0.4	42.1 ± 0.4
[P5555]Br	31.5 ± 0.4	–17.4 ± 1.3	–	–	–	46.9 ± 0.7 (upon heating)	8.91 ± 0.70 (upon heating)	63.7 ± 0.4	10.6 ± 0.6
[P5555][NTf$_2$]	21.6 ± 1.0	–14.3 ± 0.2	–	–	–	–84.6 ± 0.5 (upon cooling);<br>–82.2 ± 0.2 (upon heating)	–4.95 ± 0.02 (upon cooling);<br>5.09 ± 0.03 (upon heating)	45.7 ± 0.2	14.8 ± 0.1
[P5555][B(CN)$_4$]	39.2 ± 1.0	–9.1 ± 0.3	–	–	–	15.9 ± 0.5 (upon cooling);<br>–57.6 ± 0.4 (upon cooling);<br>–54.7 ± 0.3 (upon heating);<br>–31.4 ± 0.6 (upon heating);<br>36.8 ± 0.6 (upon heating)	–2.77 ± 0.01 (upon cooling);<br>–8.13 ± 0.11 (upon cooling);<br>8.28 ± 0.02 (upon heating);<br>–7.02 ± 0.33 (upon heating);<br>7.27 ± 0.09 (upon heating);	109.6 ± 0.3	11.1 ± 0.2
[P5555][BF$_4$]	50.7 ± 1.9	–32.6 ± 0.4	–	–	–	54.8 ± 1.1 (upon heating)	19.3 ± 0.2 (upon heating)	85.4 ± 0.2	13.3 ± 0.1
[P5555][PF$_6$]	49.2 ± 1.5	–14.7 ± 0.9	–	–	–	21.2 ± 0.6 (upon cooling);<br>32.9 ± 0.6 (upon heating)	–19.1 ± 0.1 (upon cooling);<br>19.1 ± 0.1 (upon heating)	71.2 ± 0.2	14.7 ± 1.1

2.2 Density

Density ρ was determined using a calibrated pycnometer with nominal volume of 5 mL. The ionic liquids were placed in the pycnometer and immersed in a PROLINE RP 1845 thermostat bath (LAUDA, Germany) at the stated temperature. After thermal equilibration, the sample over the mark was removed and the mass of the ionic liquid determined. From the mass and calibrated volume at the different temperatures the density was calculated. The process was repeated at different temperatures to obtain the T-dependent curves. Temperature stability during the measurements was ±0.01 K. Experimental values of the ionic liquids' densities ρ are given in Table S2. Temperature-dependent densities were fitted according to equation (S2) and are given in Table S3.

$$\rho = a - b \cdot T/\mathrm{K} \tag{S2}$$

With the linear fitting parameters a and b.

Table S2: Experimental values of the ionic liquids' temperature dependent density $\boldsymbol{\rho}$. Values are given in $\mathbf{g\ mL^{-1}}$.

Temperature /°C	$[P(3O1)_4]$ $[NTf_2]$	$[P(3O1)_4]$ $[B(CN)_4]$	$[P(3O1)_4]$ $[BF_4]$	$[P(3O1)_4]$ $[PF_6]$	[P5555] $[NTf_2]$	[P5555] $[BF_4]$	[P5555] $[PF_6]$
25	1.2861	1.0216	1.1369	1.2328	–	–	–
35	1.2780	1.0146	1.1310	1.2253	–	–	–
45	1.2694	1.0076	1.1242	1.2179	1.1403	–	–
55	1.2612	1.0011	1.1168	1.2106	1.1323	–	–
65	1.2525	0.9940	1.1105	1.2029	1.1247	–	1.0497
70	–	–	–	–	–	0.9586	1.0456
75	1.2443	0.9875	1.1040	1.1958	1.1164	0.9558	1.0426
80	–	–	–	–	–	0.9522	1.0388
85	1.2368	0.9803	1.0975	1.1882	1.1080	0.9494	1.0357
90	–	–	–	–	–	0.9458	1.0321
95	1.2276	0.9738	1.0903	1.1809	1.1005	0.9428	1.0285
100	–	–	–	–	-	0.9398	1.0244

Table S3: Linear fitting parameters $\boldsymbol{a}$ and $\boldsymbol{b}$ of the temperature-dependent density according to equation (S2) and corresponding coefficient of determination $\boldsymbol{R^2}$.

Ionic liquid	a / g mL^{-1}	b / 10^{-4} g mL^{-1} K^{-1}	R^2
$[P(3O1)_4][NTf_2]$	1.5341 ± 0.0017	8.3209 ± 0.0513	0.99977
$[P(3O1)_4][B(CN)_4]$	1.2252 ± 0.0009	6.8346 ± 0.0257	0.99992
$[P(3O1)_4][BF_4]$	1.3361 ± 0.0017	6.6702 ± 0.0516	0.99964
$[P(3O1)_4][PF_6]$	1.4532 ± 0.0004	7.4113 ± 0.0132	0.99998
$[P5555][NTf_2]$	1.3952 ± 0.0027	8.0081 ± 0.0775	0.99963
$[P5555][BF_4]$	1.1771 ± 0.0028	6.3623 ± 0.0788	0.99923
$[P5555][PF_6]$	1.2894 ± 0.0033	7.0917 ± 0.092	0.99900

2.3 Viscosity

Dynamic viscosity η was determined by shear-dependent rheology on Physica MCR301 rheometer (Anton Paar, Austria) under inert gas flow using a setup with cone-plate geometry (cone diameter of 50 mm). After thermal equilibration, the viscosity was measured with shear rates ranging from 50 to 150 s^{-1}. As there were no shear-rate or time-dependent effects observable (Newtonian flow) the viscosity values for each temperature were averaged. The process was repeated in 5 K steps to construct the *T*-dependent viscosity curves. For the samples with the $[P5555]^+$ cations the viscosity measurements were conducted starting from 378.15 K with subsequent lowering by 5 K. Temperature stability during the measurements was better than ±0.01 K. An uncertainty or the viscosity values of ±1.5% was estimated by comparison to commercial, *T*-dependent standards, repeated measurements, and literature values for established imidazolium-based ionic liquids.

Experimental values of the viscosity η are given in Table S4 and are plotted in Figure S2. The temperature-dependent viscosities were fitted with the Vogel-Fulcher-Tammann (VFT) equation (S3) with the material dependent parameters η_0, B_η and $T_{0,\eta}$.

$$\eta = \eta_0 \cdot \exp\left(\frac{B_\eta}{T - T_{0,\eta}}\right) \quad \text{(S3)}$$

The VFT fitting parameters for the viscosity data are summarized in Table S5.

Table S4: Experimental viscosity values of the ionic liquids given in mPa s.

Temperature /°C	$[P(3O1)_4]$ $[NTf_2]$	$[P(3O1)_4]$ $[B(CN)_4]$	$[P(3O1)_4]$ $[BF_4]$	$[P(3O1)_4]$ $[PF_6]$	[P5555] $[NTf_2]$	[P5555] $[BF_4]$	[P5555] $[PF_6]$
25	91.43	65.61	356.4	469.2	219.9	–	–
30	70.32	50.70	259.40	340.0	169.4	–	–
35	55.29	40.00	193.20	251.4	132.2	–	–
40	44.16	32.16	146.89	190.0	104.6	–	–
45	35.83	26.21	113.67	146.2	84.03	–	–
50	29.49	21.68	89.15	114.0	68.34	–	–
55	24.59	18.25	70.99	90.29	56.24	–	–
60	20.65	15.54	57.42	72.74	46.69	–	–
65	17.60	13.36	46.95	59.28	39.16	181.4	–
70	15.14	11.59	38.75	48.76	33.17	143.3	124.2
75	13.10	10.17	32.41	40.64	28.36	114.9	101.3
80	11.48	9.00	27.46	34.30	24.29	92.65	83.42
85	10.11	8.03	23.40	29.14	21.16	75.82	69.21
90	8.97	7.22	20.08	25.01	17.87	62.86	57.86
95	7.98	6.54	17.40	21.62	16.37	52.14	48.59
100	7.16	5.98	15.23	18.85	14.56	43.66	41.04
105	6.47	5.49	13.39	16.54	12.69	36.41	34.73

Table S5: VFT fitting parameters η_0, B_η and $T_{0,\eta}$ and coefficient of determination R^2 for the temperature-dependent viscosity η.

Ionic liquid	η_0 / 10^{-1} mPa s	B_η / K	$T_{0,\eta}$ / K	R^2
$[P(3O1)_4][NTf_2]$	1.030 ± 0.019	848.2 ± 5.2	173.2 ± 0.4	> 0.99999
$[P(3O1)_4][B(CN)_4]$	1.800 ± 0.076	640.1 ± 10.8	189.6 ± 1.1	0.99999
$[P(3O1)_4][BF_4]$	0.539 ± 0.015	1176.3 ± 8.4	164.4 ± 0.5	> 0.99999
$[P(3O1)_4][PF_6]$	0.575 ± 0.023	1209.6 ± 11.8	163.4 ± 0.7	> 0.99999
$[P5555][NTf_2]$	0.521 ± 0.040	1282.0 ± 26.2	144.6 ± 1.7	0.99999
$[P5555][BF_4]$	–	–	–	–
$[P5555][PF_6]$	–	–	–	–

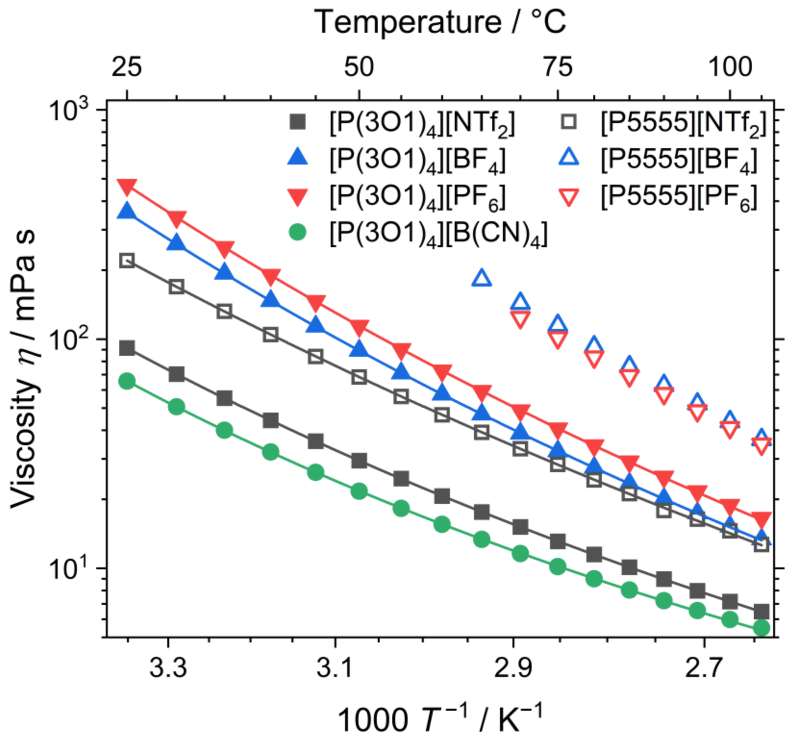

Figure S2: Experimental viscosities η at different temperatures. The drawn lines are the VFT fits according to equation (S3).

2.4 Specific Conductivity

The specific conductivity κ was measured using a SP-150 potentiostat (Biologic. France) and a commercial probe (WTW, Germany) with nominal cell constant of 0.5 cm^{-1}. The probe consisted of two rectangular platinum-electrodes in parallel arrangement which were fused in glass and freshly platinized before the measurements. The actual cell constant was determined using commercial conductivity standards. The ionic liquids were filled into the conductivity cell under argon, the cell was sealed, and immersed into a PROLINE 1845 thermostat bath (LAUDA, Germany) and after temperature-equilibration three impedance measurements were conducted with applied voltage amplitudes of 5, 10 and 15 mV and frequencies from 200 kHz to 1 Hz in 50 logarithmic steps. The electrolyte resistances determined from the three impedance measurements were averaged for each temperature. The specific conductivities were calculated from the determined resistances. The process was repeated in steps of 5 K to construct the *T*-dependent curves of the specific conductivity. The temperature stability during the measurements was better than ±0.01 K. An uncertainty or the specific conductivity measurements of ±2% was estimated by comparison to commercial conductivity standards and repeated measurements. Experimental values of the specific conductivity $\boldsymbol{\kappa}$ are given in Table S6. Temperature-dependent specific conductivity was fitted with the VFT equation (S4) with the material dependent parameters $\boldsymbol{\kappa_0}$, $\boldsymbol{B_\kappa}$ and $\boldsymbol{T_{0,\kappa}}$. VFT fitting parameters for the specific conductivity are given in Table S7 and are plotted in Figure S3.

$$\kappa = \kappa_0 \cdot \exp\left(\frac{B_\kappa}{T - T_{0,\kappa}}\right) \tag{S4}$$

Table S6: Experimental values of the ionic liquids' specific conductivity given in $\mathrm{mS\ cm^{-1}}$.

Temperature /°C	$[P(3O1)_4]$ $[NTf_2]$	$[P(3O1)_4]$ $[B(CN)_4]$	$[P(3O1)_4]$ $[BF_4]$	$[P(3O1)_4]$ $[PF_6]$	[P5555] $[NTf_2]$	[P5555] $[BF_4]$	[P5555] $[PF_6]$
25	0.893	1.574	0.332	0.259	0.323	–	–
30	1.137	1.986	0.446	0.346	0.415	–	–
35	1.417	2.462	0.586	0.460	0.526	–	–
40	1.740	3.014	0.758	0.603	0.660	–	–
45	2.115	3.632	0.966	0.777	0.814	–	–
50	2.528	4.306	1.212	0.985	0.995	–	–
55	3.002	5.036	1.494	1.231	1.200	–	0.426
60	3.523	5.838	1.828	1.512	1.432	–	0.535
65	4.088	6.716	2.203	1.836	1.692	–	0.668
70	4.689	7.651	2.635	2.207	1.982	–	0.817
75	5.334	8.640	3.118	2.631	2.304	–	1.006
80	6.026	9.695	3.663	3.107	2.661	–	1.206
85	6.783	10.806	4.262	3.640	3.054	1.082	1.437
90	7.575	11.966	4.897	4.221	3.480	1.290	1.697
95	8.415	13.174	5.587	4.834	3.946	1.532	1.965
100	9.285	14.424	6.353	5.516	4.428	1.797	2.287

Table S7: VFT fitting parameters κ_0, B_κ and $T_{0,\kappa}$ and coefficient of determination R^2 for the temperature-dependent specific conductivity κ.

Ionic liquid	κ_0 / mS cm^{-1}	B_κ / K	$T_{0,\kappa}$ / K	R^2
$[P(3O1)_4][NTf_2]$	411.7 ± 11.6	−743.7 ± 9.4	177.0 ± 1.0	0.99999
$[P(3O1)_4][B(CN)_4]$	408.6 ± 5.2	−628.7 ± 4.0	185.1 ± 0.5	> 0.99999
$[P(3O1)_4][BF_4]$	984.1 ± 65	−1019 ± 23	171.0 ± 2.0	0.99999
$[P(3O1)_4][PF_6]$	1031.6 ± 78.2	−1055 ± 27	171.5 ± 2.2	0.99998
$[P5555][NTf_2]$	771.3 ± 32.3	−1147.7 ± 16.4	150.7 ± 1.4	> 0.99999
$[P5555][BF_4]$	–	–	–	–
$[P5555][PF_6]$	–	–	–	–

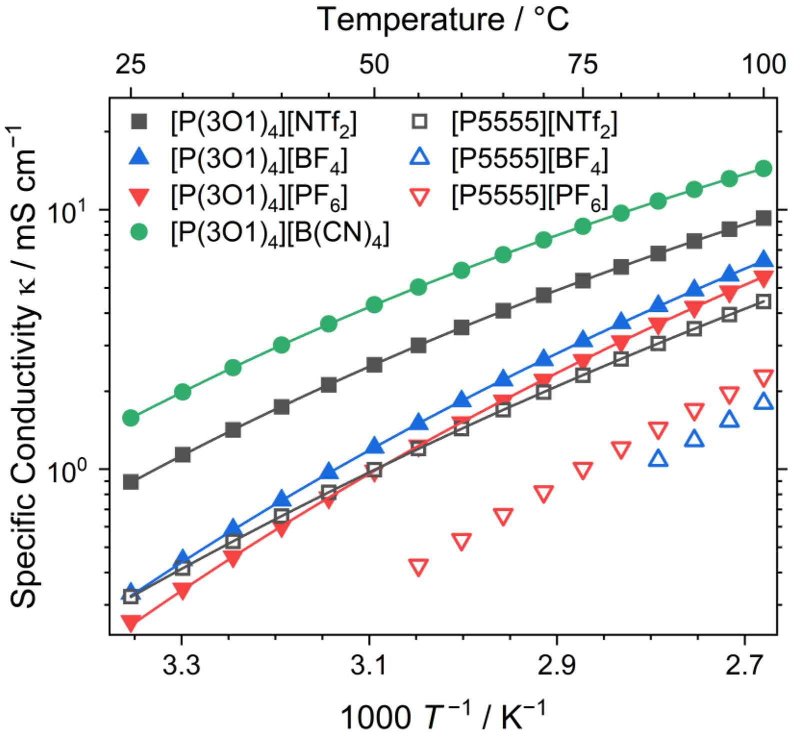

Figure S3: Experimental specific conductivities κ at different temperatures. The drawn lines are the VFT fits according to equation (S4).

2.5 Molar Conductivity

Molar conductivity Λ_M was calculated from the experimental specific conductivities and the linear fits of the experimental density and molar mass M according to equation (S5).

$$\Lambda_\mathrm{M} = \frac{\kappa \cdot M}{\rho} \tag{S5}$$

The resulting values for the molar conductivity are given in Table S8 and are plotted in Figure S4. The temperature-dependent molar conductivity was fitted with the VFT-equation (S6), with $\Lambda_\mathrm{M,0}$, B_{Λ_M} and T_{0,Λ_M} being material dependent parameters, as summarized in Table S9.

$$\Lambda_\mathrm{M} = \Lambda_\mathrm{M,0} \cdot \exp\left(\frac{B_{\Lambda_\mathrm{M}}}{T - T_{0,\Lambda_\mathrm{M}}}\right) \tag{S6}$$

Table S8: Calculated values of the ionic liquids' molar conductivity given in $\mathrm{S\ cm^2\ mol^{-1}}$.

Temperature /°C	$[P(3O1)_4]$ $[NTf_2]$	$[P(3O1)_4]$ $[B(CN)_4]$	$[P(3O1)_4]$ $[BF_4]$	$[P(3O1)_4]$ $[PF_6]$	[P5555] $[NTf_2]$	[P5555] $[BF_4]$	[P5555] $[PF_6]$
25	0.419	0.676	0.128	0.098	0.093	–	–
30	0.536	0.855	0.172	0.132	0.120	–	–
35	0.669	1.064	0.227	0.176	0.153	–	–
40	0.825	1.306	0.295	0.231	0.192	–	–
45	1.005	1.580	0.377	0.299	0.238	–	–
50	1.206	1.879	0.474	0.380	0.292	–	–
55	1.437	2.205	0.586	0.476	0.353	–	–
60	1.692	2.565	0.719	0.587	0.423	–	–
65	1.969	2.961	0.869	0.715	0.502	–	0.192
70	2.267	3.385	1.043	0.862	0.590	–	0.235
75	2.587	3.836	1.238	1.031	0.688	–	0.291
80	2.932	4.319	1.459	1.221	0.797	–	0.350
85	3.312	4.831	1.702	1.435	0.918	0.314	0.418
90	3.711	5.368	1.962	1.669	1.050	0.375	0.495
95	4.137	5.931	2.245	1.917	1.195	0.447	0.576
100	4.580	6.516	2.561	2.195	1.346	0.526	0.672

Table S9: VFT fitting parameters $\Lambda_\mathrm{M,0}$, B_{Λ_M} and T_{0,Λ_M} and coefficient of determination R^2 for the temperature-dependent molar conductivity Λ_M.

Ionic liquid	$\Lambda_\mathrm{M,0}$ / $\mathrm{S\ cm^2\ mol^{-1}}$	B_{Λ_M} / K	T_{0,Λ_M} / K	R^2
$[P(3O1)_4][NTf_2]$	249.3 ± 7.4	–800.0 ± 10.2	173.0 ± 1.1	0.99999
$[P(3O1)_4][B(CN)_4]$	225.3 ± 2.9	–680.8 ± 4.1	181.0 ± 0.5	> 0.99999
$[P(3O1)_4][BF_4]$	480.4 ± 31.8	–1073 ± 24	168.1 ± 2.0	0.99999
$[P(3O1)_4][PF_6]$	499.4 ± 38.2	–1110 ± 27	168.6 ± 2.2	0.99999
$[P5555][NTf_2]$	305.1 ± 13.5	–1231.0 ± 17.7	146.1 ± 1.4	> 0.99999
$[P5555][BF_4]$	–	–	–	–
$[P5555][PF_6]$	–	–	–	–

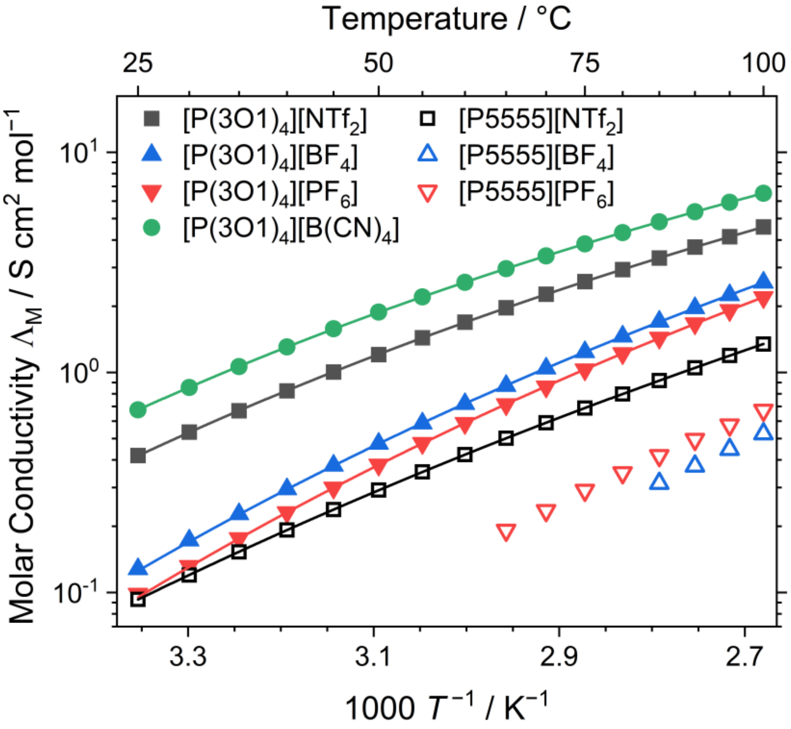

Figure S4: Calculated molar conductivities Λ_M at different temperatures. The drawn lines are the VFT fits according to equation (S6).

2.6 Self-diffusion Coefficients

Self-diffusion coefficients D_{Si} (i = +: cation or i = −: anion) were determined by the pulsed field gradient stimulated spin echo (PFGSTE) pulse sequence of the NMR spectroscopy with bipolar gradients and longitudinal eddy current delay. Therefore, the samples were places in the insert of coaxial NMR tubes (diameter ≈1 mm) under argon atmosphere and the tubes were evacuated and flame sealed to avoid the uptake of atmospheric moisture. The narrow sample geometry was chosen to minimize convection. The self-diffusion coefficients were measured on an Avance Neo 500 spectrometer (Bruker, USA) equipped with a Prodigy TCI cryo probe head and a BCU II temperature unit. The temperature control of the spectrometer was calibrated with methanol and ethylene glycol[4] and the gradient unit was checked by measurements of pure water and established ionic liquids. Cation self-diffusion coefficients were determined using the signals of the ^{1}H nucleus, while for the anion self-diffusion coefficients the ^{19}F nucleus was used. After temperature equilibration the pulse width and longitudinal relaxation time T_1 were determined. Parameters for the diffusion time Δ and the duration of the magnetic field δ were chosen to give a signal attenuation of 95% when gradient strengths g of 2 to 95% of the maximum gradient strength (65.7 G cm^{-1}) are used. The gradient shape used was that of smoothed rectangular. With the optimized parameters the actual measurement consisting of 16 measurements, each with 16 scans, with linearly increased gradient strength (from 2 to 95% of the maximum gradient strength), were conducted. The self-diffusion coefficients were obtained by regression of the Stejskal-Tanner equation (S7),

$$I = I_0 \cdot \exp\left(-D_{\mathrm{S}i}(\gamma g \delta)^2(\Delta - \delta/3)\right) \qquad \text{(S7)}$$

With I the signal intensity of the measurements with applied magnetic field gradients, I_0 the initial signal intensity, ⍰ the gyromagnetic ratio of the investigated nucleus, g the gradient strength of the applied magnetic field gradient. An uncertainty for the self-diffusion measurements of ±2% was estimated by comparison to literature values of reference samples, repeated measurements, and variation of experimental parameters.

Experimental self-diffusion coefficients $D_{\mathrm{S}i}$ ($i = +$: cation, $i = -$: anion) as obtained by the pulsed field gradient stimulated spin echo of the nuclear magnetic resonance spectroscopy are given in Table S10. Table S10 and Table S11 and are plotted in Figure S5 and Figure S6.

The temperature-dependent self-diffusion coefficients were fitted with the VFT equation (S8) with $D_{\mathrm{S}i,0}$, $B_{D_{\mathrm{S}i}}$ and $T_{0,D_{\mathrm{S}i}}$ being material dependent fitting parameters. The results are summarized in Table S12.

$$D_{\mathrm{S}i} = D_{\mathrm{S}i,0} \cdot \exp\left(\frac{B_{D_{\mathrm{S}i}}}{T - T_{0,D_{\mathrm{S}i}}}\right) \qquad \text{(S8)}$$

Table S10: Experimental values of the ionic liquids' cation self-diffusion coefficients D_{S+} given in $m^2\ s^{-1}$ as obtained by ^{1}H NMR spectroscopy.

Temperature /°C	$[P(3O1)_4]$ $[NTf_2]$	$[P(3O1)_4]$ $[B(CN)_4]$	$[P(3O1)_4]$ $[BF_4]$	$[P(3O1)_4]$ $[PF_6]$	[P5555] $[NTf_2]$	[P5555] $[BF_4]$	[P5555] $[PF_6]$
5	2.73E−12	3.08E−12	–	–	–	–	–
10	3.91E−12	4.50E−12	–	–	–	–	–
15	5.44E−12	6.35E−12	1.07E−12	–	–	–	–
20	7.42E−12	8.76E−12	1.56E−12	1.25E−12	–	–	–
25	9.88E−12	1.17E−11	2.21E−12	1.79E−12	3.30E−12	–	–
30	1.29E−11	1.54E−11	3.08E−12	2.52E−12	4.38E−12	–	–
35	1.65E−11	1.98E−11	4.18E−12	3.47E−12	5.72E−12	–	–
40	2.09E−11	2.51E−11	5.63E−12	4.69E−12	7.40E−12	–	–
45	2.59E−11	3.11E−11	7.39E−12	6.20E−12	9.35E−12	–	–
50	3.17E−11	3.80E−11	9.54E−12	8.15E−12	1.18E−11	–	–
55	3.83E−11	4.60E−11	1.21E−11	1.04E−11	1.45E−11	3.35E-12	4.05E−12
60	4.55E−11	5.48E−11	1.50E−11	1.31E−11	1.77E−11	4.40E-12	5.22E−12
65	5.42E−11	6.46E−11	1.86E−11	1.62E−11	2.14E−11	5.70E-12	6.69E−12
70	6.32E−11	7.53E−11	2.28E−11	1.99E−11	2.57E−11	7.30E-12	8.39E−12
75	7.36E−11	8.72E−11	2.76E−11	2.40E−11	3.06E−11	9.23E-12	1.04E−11
80	8.46E−11	9.99E−11	3.30E−11	2.89E−11	3.60E−11	1.16E-11	1.28E−11
85	9.66E−11	1.14E−10	3.92E−11	3.44E−11	4.21E−11	1.43E-11	1.57E−11
90	1.10E−10	1.29E−10	4.61E−11	4.07E−11	4.89E−11	1.77E-11	1.91E−11

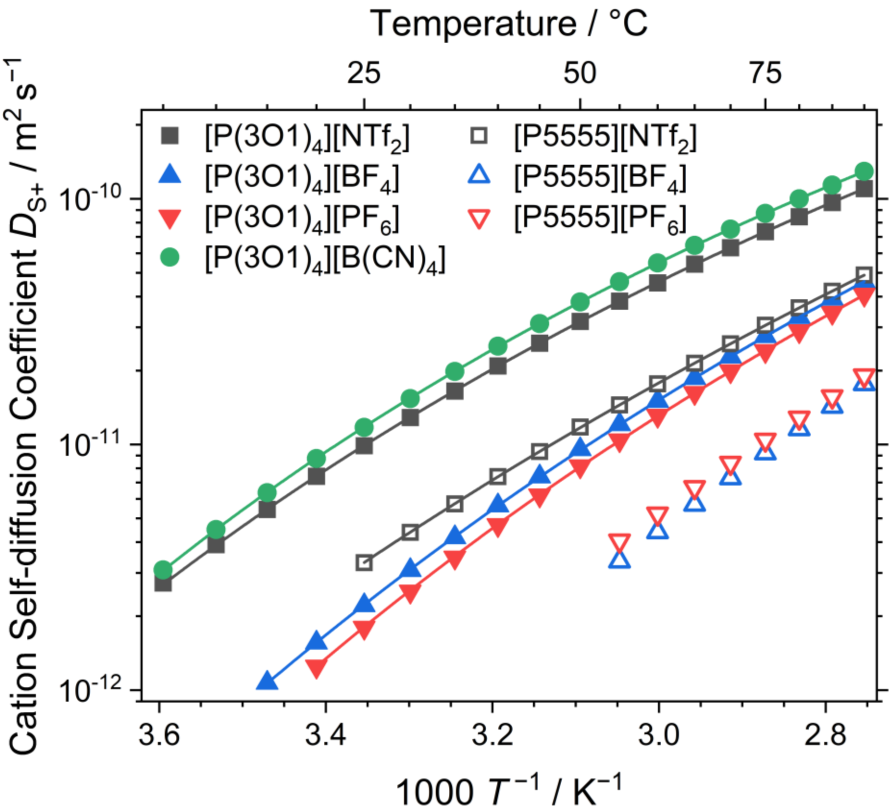

Figure S5: Experimental cation self-diffusion coefficients $\boldsymbol{D_{S+}}$ at different temperatures. The drawn lines are the VFT fits according to equation (S8).

Table S11: Experimental values of the ionic liquids' cation self-diffusion coefficients $\boldsymbol{D}_{\mathbf{S-}}$ given in $\mathbf{m^2\ s^{-1}}$ as obtained by ^{19}F NMR spectroscopy.

Temperature /°C	$[P(3O1)_4]$ $[NTf_2]$	$[P(3O1)_4]$ $[B(CN)_4]$	$[P(3O1)_4]$ $[BF_4]$	$[P(3O1)_4]$ $[PF_6]$	[P5555] $[NTf_2]$	[P5555] $[BF_4]$	[P5555] $[PF_6]$
5	3.34E−12	–	–	–	–	–	–
10	4.79E−12	–	–	–	–	–	–
15	6.68E−12	–	1.76E−12	–	–	–	–
20	9.12E−12	–	2.57E−12	1.83E−12	–	–	–
25	1.21E−11	–	3.64E−12	2.66E−12	4.69E−12	–	–
30	1.58E−11	–	5.07E−12	3.75E−12	6.24E−12	–	–
35	2.03E−11	–	6.90E−12	5.19E−12	8.16E−12	–	–
40	2.56E−11	–	9.25E−12	7.02E−12	1.05E−11	–	–
45	3.18E−11	–	1.21E−11	9.29E−12	1.34E−11	–	–
50	3.87E−11	–	1.56E−11	1.22E−11	1.67E−11	–	–
55	4.70E−11	–	1.97E−11	1.56E−11	2.07E−11	4.50E-12	5.34E−12
60	5.60E−11	–	2.47E−11	1.96E−11	2.54E−11	5.94E-12	6.89E−12
65	6.65E−11	–	3.06E−11	2.45E−11	3.07E−11	7.72E-12	8.84E−12
70	7.76E−11	–	3.74E−11	3.00E−11	3.69E−11	9.90E-12	1.12E−11
75	9.00E−11	–	4.51E−11	3.63E−11	4.37E−11	1.25E-11	1.39E−11
80	1.04E−10	–	5.42E−11	4.37E−11	5.15E−11	1.57E-11	1.72E−11
85	1.19E−10	–	6.42E−11	5.22E−11	6.04E−11	1.94E-11	2.11E−11
90	1.35E−10	–	7.57E−11	6.16E−11	7.02E−11	2.39E-11	2.57E−11

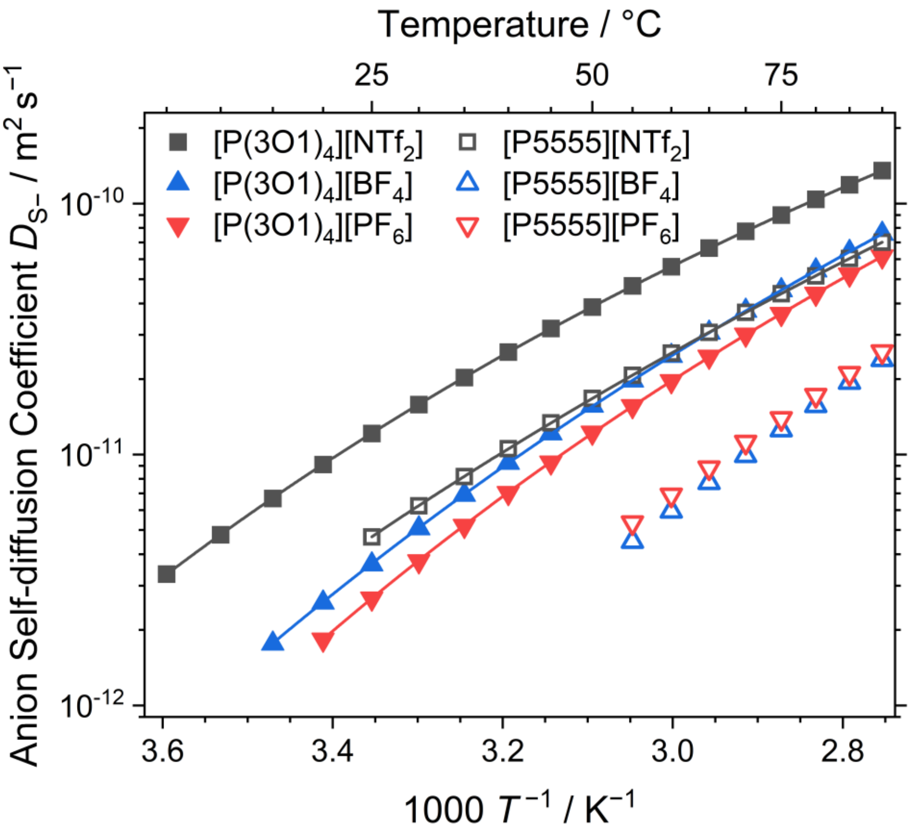

Figure S6: Experimental anion self-diffusion coefficients $\boldsymbol{D}_{\mathbf{S-}}$ at different temperatures. The drawn lines are the VFT fits according to equation (S8).

Table S12: VFT fitting parameters $\boldsymbol{D_{Si,0}}$, $\boldsymbol{B_{D_{Si}}}$ and $\boldsymbol{T_{0,D_{S,i}}}$ and coefficient of determination $\boldsymbol{R^2}$ for the temperature-dependent self-diffusion coefficients $\boldsymbol{D_{Si}}$.

Ionic liquid	i	D_{si} / 10^{-8} m^2 s^{-1}	$B_{D_{si}}$ / K	$T_{0,D_{si}}$ / K	R^2
$[P(3O1)_4][NTf_2]$	+	1.447 ± 0.066	−960.6 ± 15.5	166.3 ± 1.4	0.99999
$[P(3O1)_4][NTf_2]$	–	1.895 ± 0.065	−981.0 ± 11.8	164.7 ± 1.0	>0.99999
$[P(3O1)_4][B(CN)_4]$	+	1.167 ± 0.053	−844.0 ± 14.5	175.8 ± 1.4	0.99999
$[P(3O1)_4][BF_4]$	+	2.979 ± 0.142	−1319.4 ± 17.3	159.3 ± 1.2	>0.99999
$[P(3O1)_4][BF_4]$	–	5.395 ± 0.305	−1354.4 ± 20.8	157.0 ± 1.4	>0.99999
$[P(3O1)_4][PF_6]$	+	2.359 ± 0.285	−1259.1 ± 42.6	165.3 ± 3.0	0.99998
$[P(3O1)_4][PF_6]$	–	3.468 ± 0.312	−1242.8 ± 31.5	166.9 ± 2.2	0.99999
$[P5555][NTf_2]$	+	3.130 ± 0.198	−1426.9 ± 25.1	142.3 ± 1.7	>0.99999
$[P5555][NTf_2]$	–	4.512 ± 0.280	−1425.7 ± 24.6	142.7 ± 1.7	>0.99999
$[P5555][BF_4]$	+	29.60 ± 14.21	−2337.8 ± 217.8	122.8 ± 10.5	0.99998
$[P5555][BF_4]$	–	20.69 ± 6.56	−2045.2 ± 134.4	137.6 ± 7.0	0.99999
$[P5555][PF_6]$	+	14.46 ± 6.83	−2123.7 ± 211.3	125.5 ± 11.1	0.99998
$[P5555][\ PF_6]$	–	23.30 ± 12.23	−2174.4 ± 235.9	124.6 ± 12.2	0.99998

3. Ab initio simulations

The Gaussian 09 software package, Revision E.01, was used for all *ab initio* simulations.[5] Energies and atomic charges for force field parameterization were obtained at the full MP2/cc-pVTZ//B3LYP-GD3BJ/6-311+G(d,p) level of theory. A pruned integration grid with 99 radial shells and 590 angular points per shell was used. The SCF convergence criteria were 10^{-11} RMS change and 10^{-8} maximum change in the density matrix for MP2 single point calculations. For DFT calculations, the SCF convergence criterium was 10^{-10} RMS change and 10^{-9} maximum change in the density matrix.

To ensure that curling is present in the cation chosen for the experimental study, we investigated the effect of the position of the ether group in the side chain. To this end, three model systems were used with the ether oxygen in β, γ or δ position, i.e. $[P111(1O3)]^+$, $[P111(2O2)]^+$ and $[P111(3O1)]^+$, respectively. The $[P1115]^+$ cation was also included for comparison.

Figure S7 shows the potential energy surface corresponding to rotation around the P-C bond. In general, the differences were marginal. The picture was similar for the dihedral at the other end of the side chain, Figure S8. It is worth noting that the $[P111(2O2)]^+$ cation showed the lowest energy barriers for the anticlinal transition state, which is equivalent to a higher conformational flexibility.

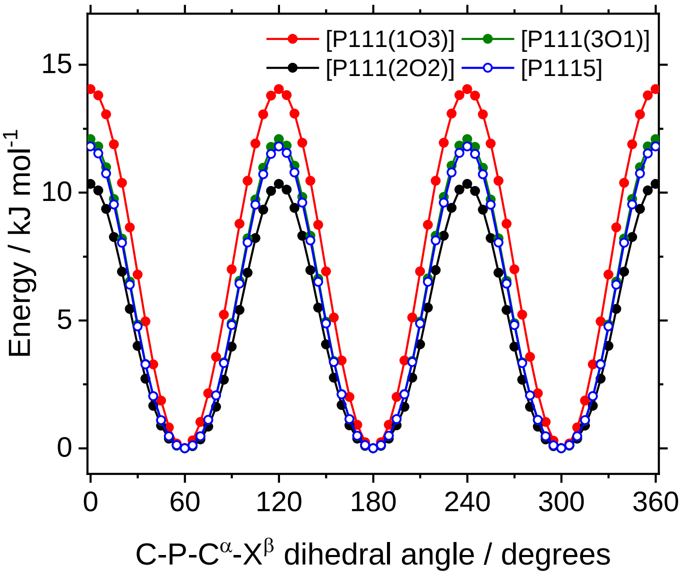

Figure S7: The energy as a function of the C-P-C^α-X^β dihedral angle, with X = CH_2,O.

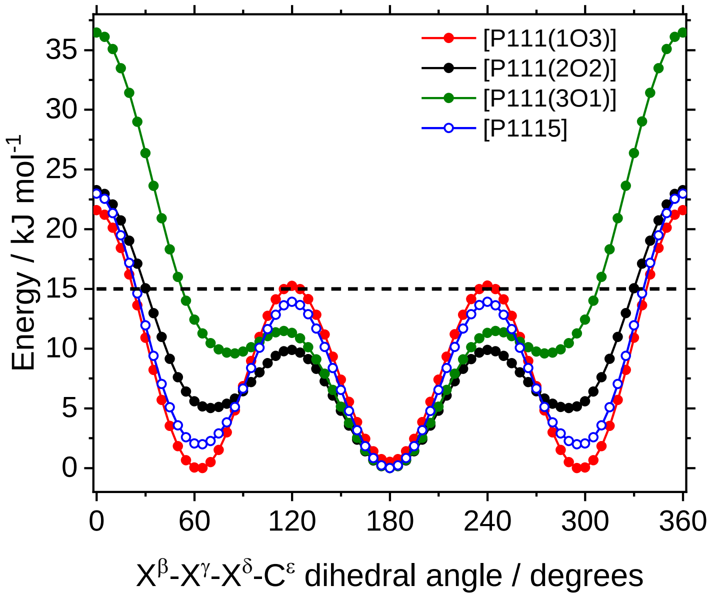

Figure S8: The energy as a function of the X^{β}-X^{γ}-X^{δ}-C^{ε} dihedral angle, with X = CH_2,O.

The rotation around the bond between the α carbon and the atom in β position was observed to be the defining characteristic of the conformational space of the $[P111(2O1)]^+$ and $[N111(2O1)]^+$ cations.[2] This was also the case for $[P111(2O2)]^+$, Figure S9. However, other positions of the ether oxygen, i.e. those in the $[P111(3O1)]^+$ and $[P111(1O3)]^+$ cations, did not lead to the typical global minimum near 60°. However, these one-dimensional potential energy profiles are traces of a much more complicated high dimensional potential energy surface (*cf.* Figure S11) where the partially constrained optimization was initialized with all other dihedrals set to 180°. For $[P111(1O3)]^+$, the gauche structure was not preferred since the distance between phosphonium center and the oxygen atom does not change in this cation, regardless of its conformation. In contrast, in order to minimize the distance between phosphorus and oxygen for $[P111(3O1)]^+$, the dihedral angle corresponding to rotation around the bond between β and γ atoms must take values of ≈ 60° or ≈ 300°. The clear preference for the gauche conformer in the C-C-C-O dihedral angle in $[P111(3O1)]^+$ compared to the other cations is shown in Figure S10.

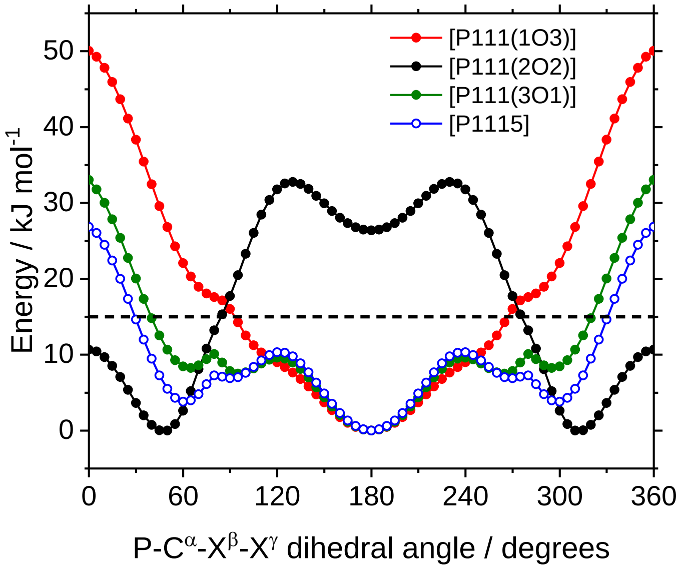

Figure S9: The energy as a function of the P-C^{α}-X^{β}-X^{γ} dihedral angle, with X = CH_2,O.

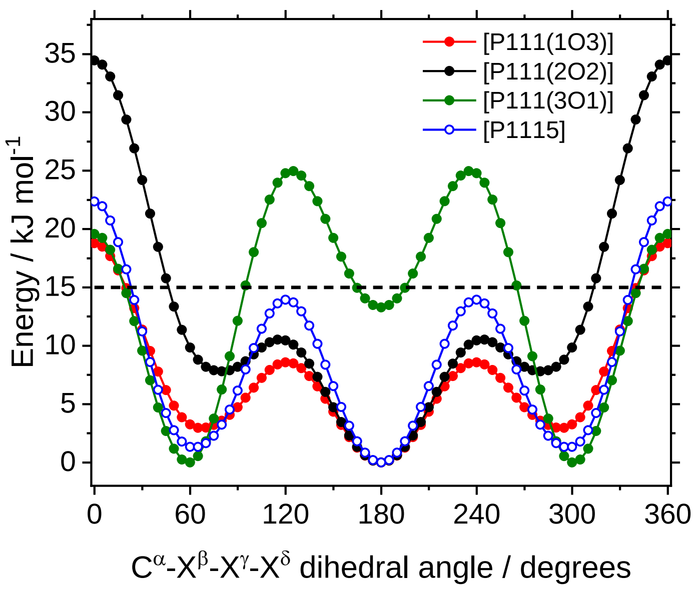

Figure S10: The energy as a function of the C^{α}-X^{β}-X^{γ}-X^{δ} dihedral angle, with X = CH_2,O.

The unique behavior of the $[P111(3O1)]^+$ cation due to the three methylene groups separating phosphorus atom and ether oxygen becomes apparent from the two dimensional potential energy surface, Figure S11. The dihedral angles in Figure S11 correspond to rotation around the two C-C bonds in the cation. The four conformers which were considered relevant are labelled GS1-GS4 in Figure S11, in line with the main manuscript. The energy profiles of the two dihedral angles were not independent. Thus, starting the partially constrained optimization of the P-C-C-C dihedral angle with the C-C-C-O dihedral angle initialized in a gauche conformation led to a more realistic profile, considerably lowered in energy compared to the one obtained for an initial antiperiplanar conformation of the C-C-C-O dihedral angle, Figure S12. For better comparison with Fig. 3 in the main manuscript, the energies of the GS1-4 are shown in Table S13 for $[P111(3O1)]^+$, $[P1115]^+$ fixed (geometry optimized with partial restraints on the two dihedrals), and $[P1115]^+$ free (geometry optimized without restraints).

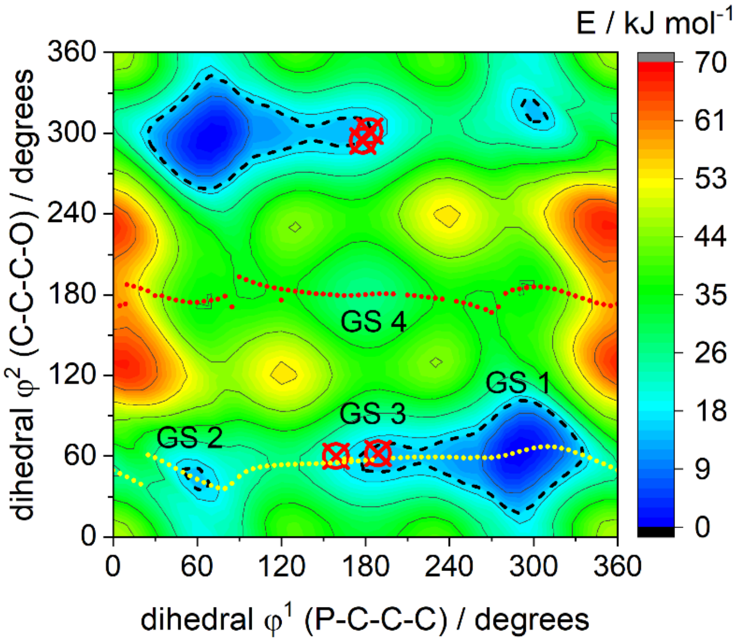

Figure S11: The two dimensional potential energy surface for the $[P111(3O1)]^+$ cation. The red crossed circles indicate the geometry in the crystal structures. For the dotted lines see Figure S12.

Table S13: The energies of the four relevant conformers of the $[P111(3O1)]^+$ model system. For comparison, the energies of the corresponding alkyl side chain were also calculated using GS1-4 as starting structures but replacing O with CH_2. Two calculations were performed: a partial relaxation where the geometry was optimized while keeping the two dihedrals ϕ_1 and ϕ_2 were frozen ("Fixed"), and a full, unconstrained geometry optimization ("Free").

	Energy / kJ mol^{-1}		
	$[P111(3O1)]^+$	$[P1115]^+$	
		Fixed	Free
GS1	0.0	23.3	13.6
GS2	13.8	9.6	3.3
GS3	14.2	3.2	1.3
GS4	27.5	0.0	0.0

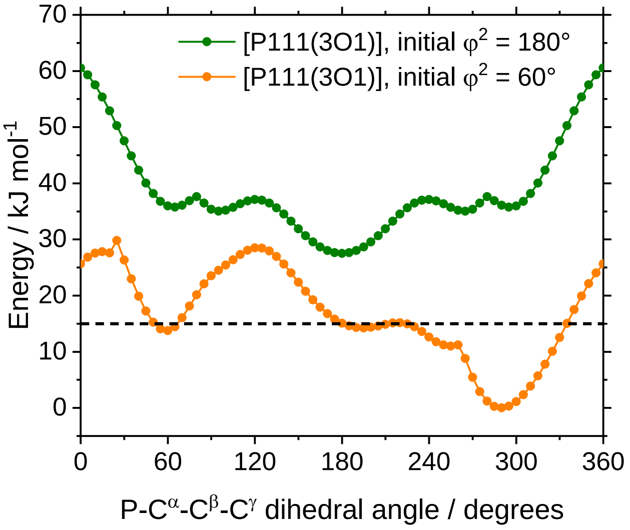

Figure S12: Two partially relaxed scans of the P-C-C-C dihedral angle with the C-C-C-O dihedral angle initialized to either 180° or 60°. For comparison, the geometries corresponding to the orange (green) curve are marked with yellow (red) dots in Figure S11.

4. MD simulations

The LAMMPS software package version 11 August 2017, was used with periodic boundary conditions for all molecular dynamics simulations.[6] Neighbor lists were used and rebuilt with a skin distance of 2.0 Å. Coulombic interactions were calculated with a particle-particle particle-mesh solver with a relative RMS force error of 10^{-5}. Nonbonded interactions (Coulomb and 12/6 Lennard-Jones) were calculated with a cutoff of 12.0 Å, scaled by 0.0/0.0/0.5 for pairs of atoms exactly 1/2/3 bonds apart, and include a Van der Waals tail correction.[7] Geometric mixing rules were used for Lennard-Jones parameters between different atom types. The shake algorithm with a tolerance of 10^{-4} and a maximum of 20 iterations was used to constrain C-H bonds to their equilibrium value.[8] A timestep of 1.0 fs was used, the simulation temperature was set to 400 K (pressure 1 bar) to compensate for the slow dynamics of the non-polarizable force field. This choice was made since only the relative change in structure (=configurational entropy) was the desired quantity. Other quantities are given below for the sake of completeness, however quantitative predictions especially of dynamics are not reliable with this setup. The linear and angular momentum of the simulation box was subtracted every 10000 steps, including appropriate re-scaling of the velocities. The equations of motion were integrated numerically with the velocity-Verlet algorithm. A Nosé-Hoover chain thermostat (thermostat-barostat) with a chain length of three and a damping parameter of 100 fs (1000 fs) was used for NVT (NPT) simulations, including the Martyna-Tuckerman-Klein correction.[9]

The TRAVIS software package, code version 01 Jan 2019 was used to calculate radial and spatial distribution functions.[10,11] Other analyses were performed with the prealpha software package (source code, executable, input files and manual available on github, https://github.com/FPhilippi/prealpha). 3D visualization of structures was achieved with VMD.[12]

4.1 Force field development

Atomic dipole corrected Hirshfeld charges (ADCH) were calculated with Multiwfn and used for the simulation.[13,14] Charges were averaged over symmetrically identical atoms and rounded to three digits, the charges on the phosphorus atoms were adjusted to yield integer total charges on the ions ($[P(3O1)_4]^+$: from 0.403 e to 0.412 e; $[P5555]^+$: from 0.414 e to 0.408 e). Other force field parameters, *i.e.* Lennard-Jones parameters, equilibrium values and stretching constants for bonds and angles, as well as dihedral parameters were taken from the literature.[15–20] This is with the exception of the backbone dihedral parameters of the ether-functionalized cation, which were fitted as follows based on the *ab initio* simulations of the $[P111(3O1)]^+$ model system. The otherwise complete force field with zeroed backbone dihedral components was used to perform a 2D energy scan of both the P-C-C-C and C-C-C-O dihedral angles, and the difference to the *ab initio* PES (*cf.* Figure S11) was fitted in two dimensions to obtain the corresponding OPLS parameters. To obtain the OPLS parameters for the C-P-C-C and C-C-O-C dihedrals, restraints were applied to all backbone dihedrals except the one to be scanned/fitted (restraints were 60 degrees for C-P-C-C and 180 degrees for P-C-C-C, C-C-C-O, C-C-O-C). Then, the energy was calculated as a function of angle, and the difference to the *ab initio* function fitted as usual. To verify the resulting final force field, a 2D energy scan of the P-C-C-C and C-C-C-O dihedral angles was performed again, Figure S13. The resulting PES agrees well with the *ab initio* surface despite a small, artificial stabilization of GS2 and GS3.

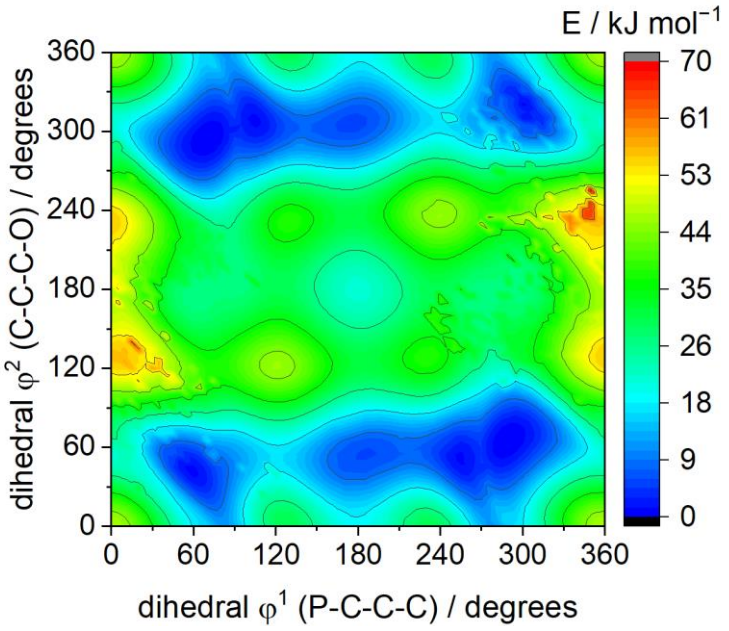

Figure S13: The two dimensional potential energy surface for the $[P111(3O1)]^+$ cation as obtained with the classical force field.

4.2 Preparation and equilibration

Initially, 1000 ion pairs were packed randomly into a box with 180 Å side length using packmol.[21] The LAMMPS data files were prepared using fftool (https://github.com/paduagroup/fftool), and the energy of the system minimized with a conjugate gradient algorithm. Atoms were then assigned random velocities according to the target temperature.

The system was kept at the target temperature of 400 K for 40 ps, heated to 600 K in a linear ramp over 10 ps, kept at 600 K for 500 ps, cooled to 400 K with a linear ramp over 10 ps, and kept at 400 K for 10 ps. This initial annealing procedure (570 ps) was performed in the NPT ensemble. A brief pre-equilibration period was performed at 400 K for 300 ps in NVT, followed by 500 ps in NPT, averaging the cell volume (recorded every 10 steps) over the NPT run. The local average was saved to a file and the simulation box compressed to the local average over 100 ps in NVT. This three-step sequence was repeated until no further drift could be observed in the local averages. The resulting global averages of the cell vectors were 90.71(3) Å for $[P5555][BF_4]$ and 86.79(2) Å for $[P(3O1)_4][BF_4]$. The simulation box was compressed to this global average over 100 ps, and equilibrated in NVT for 10 ns. This was followed by a 20 ns production run in the microcanonical ensemble, recording the atomic positions every 1 ps.

4.3 Trajectory analyses

The radial distribution functions used for the calculation of excess configurational entropy are shown in Figure S14, Figure S15, and Figure S16. Notably, the first peak in the cation-anion distribution function is shifted to larger distances for the ether-functionalized ionic liquid. For $[P(3O1)_4][BF_4]$, a secondary small peak appears around 9.3 Å in Figure S16, which coincides with a minimum for $[P5555][BF_4]$. The changes in the like-charge RDFs are significantly smaller and more of a quantitative nature. This is a signature of the cation curling, which pushes some of the anions away from the cation (from 5.5 Å to 9.3 Å).

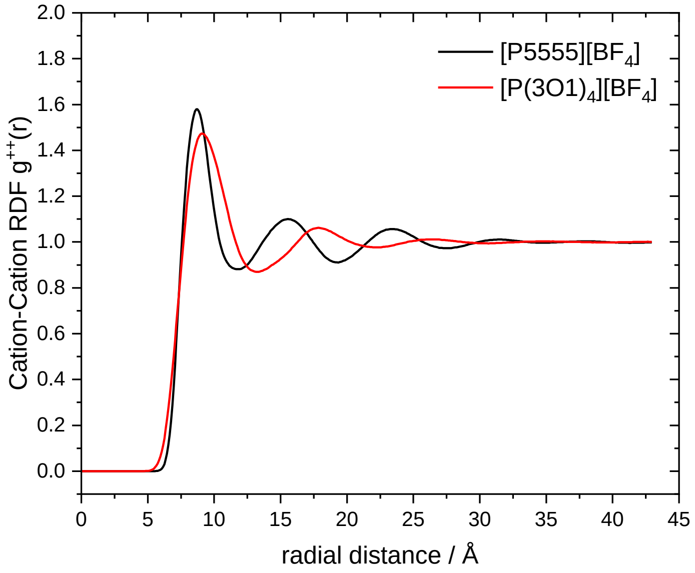

Figure S14: Cation-Cation radial distribution functions obtained from the MD simulation.

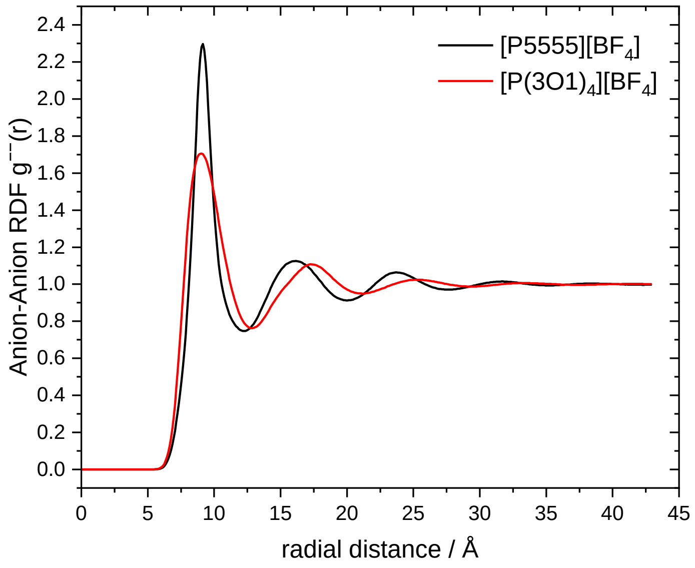

Figure S15: Anion-Anion radial distribution functions obtained from the MD simulation.

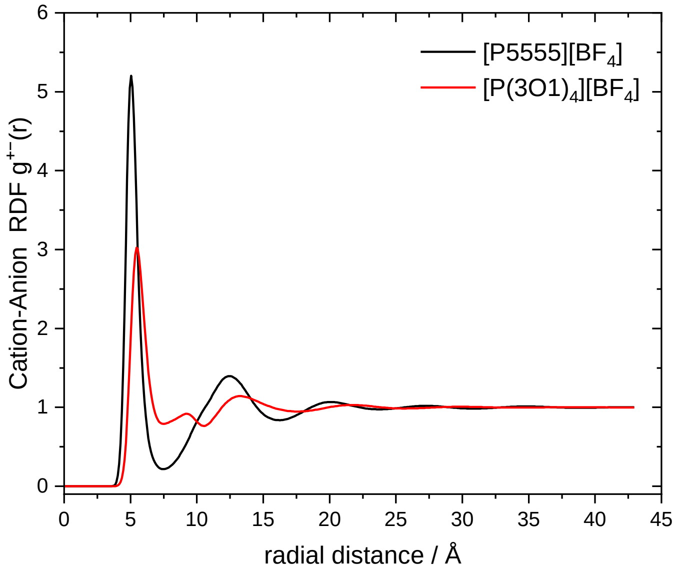

Figure S16: Cation-Anion radial distribution functions obtained from the MD simulation.

The configurational excess entropy $S_{ex,config}$ was calculated from the radial distribution functions $g(r)$ using equation (S9).[22] Three-body interactions (and higher) are not included.

$$S_{ex,config} = \frac{-R\rho}{2} \int_{0}^{\infty} 4\pi r^2 (g \ln g - g + 1) \mathrm{d}r \qquad \text{(S9)}$$

Here, R is the ideal gas constant and ρ is the number density (the number of ion pairs divided by the simulation box volume). The running integrals, equation (S10), are shown in Figure S17 and Figure S18, demonstrating convergence of the integral within approximately 30 Å. The main difference between the two systems is found in the cation-anion contribution to configurational excess entropy (and RDF), hence the contribution per radial bin to the integral is also given in Figure S19. For $[P(3O1)_4][BF_4]$, only the first shell is relevant (as well as the excluded volume at short distances). For $[P5555][BF_4]$, contributions from the shell structure to the configurational excess entropy are discernible up to a distance of >20 Å. Note that also like charge shells appear in the cation-anion contribution to configurational excess entropy.

$$S_{ex,config}(r) = \frac{-R\rho}{2} \int_{0}^{r} 4\pi r'^2 (g(r') \ln g(r') - g(r') + 1) \mathrm{d}r' \qquad \text{(S10)}$$

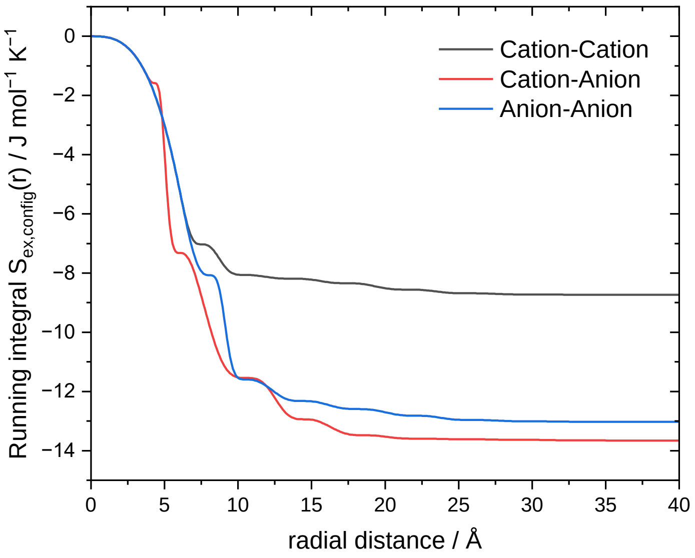

Figure S17: Running integral of the excess configurational entropy from the radial distribution functions, for the MD simulation of [P5555][BF_4].

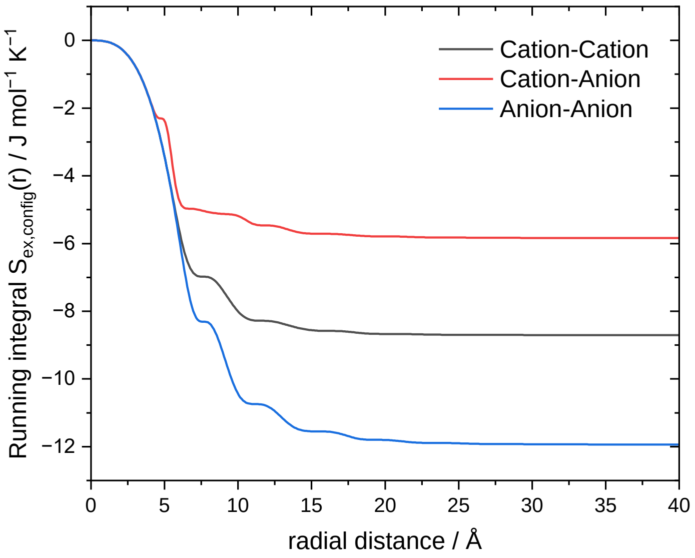

Figure S18: Running integral of the excess configurational entropy from the radial distribution functions, for the MD simulation of [P(3O1)$_4$][BF_4].

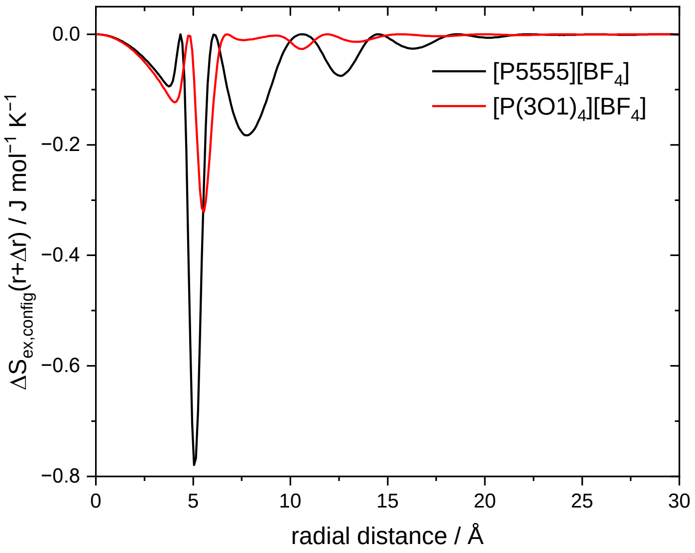

Figure S19: Contributions per radial bin to the excess configurational entropy.

It is also possible to calculate the configurational excess entropy from the spatial distribution function. In practice, the summation over voxels is used, equation (S11). The indices i, j, k specify each voxel in x, y, z direction. Note that $g(r) \approx 4\pi r^2\, g(\vec{r})$ for a system with angular isotropy. Here, the voxels have uniform cubic shape, thus the volume of each voxel $V_{voxel} = \Delta x \Delta y \Delta z$ is constant.

$$S_{ex,config}^{SDF} = \frac{-R\rho}{2} \sum_{i,j,k} \{(g_{ijk} \ln g_{ijk} - g_{ijk} + 1)\} \Delta x \Delta y \Delta z \quad \text{(S11)}$$

The spatial distribution functions in the main manuscript were created based on this equation. The absolute value of the entropy obtained in this way is necessarily more negative due to the presence of angular dependence in the structuring. However, the total value of the configurational excess entropy is more difficult to converge due to small noise in the spatial distribution function, Figure S20, which is why we chose the more robust approach based on the radial distribution.

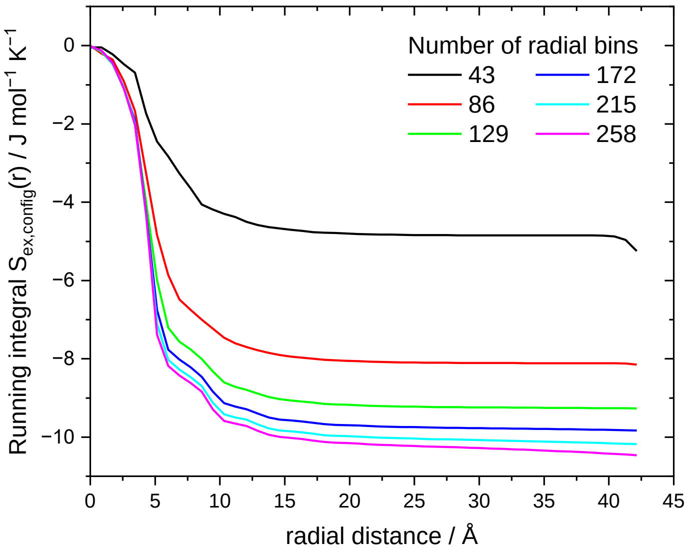

Figure S20: Configurational excess entropy running integral / sum as a function of radial distance based on the spatial distribution function of [P(3O1)$_4$][BF$_4$]. Different bin counts were used to calculate the spatial distribution function.

For comparison with Fig. 4b) in the main manuscript, the isosurface for [P5555][BF_4] encompassing a similar volume to the isosurface for [P(3O1)$_4$][BF_4] (which was drawn at a threshold of mJ mol^{-1} K^{-1} Å^{-3}) is shown in Figure S21. The threshold for the configurational excess entropy to give a similar volume is five times as high for the alkyl functionalized cation compared to the ether functionalized cation.

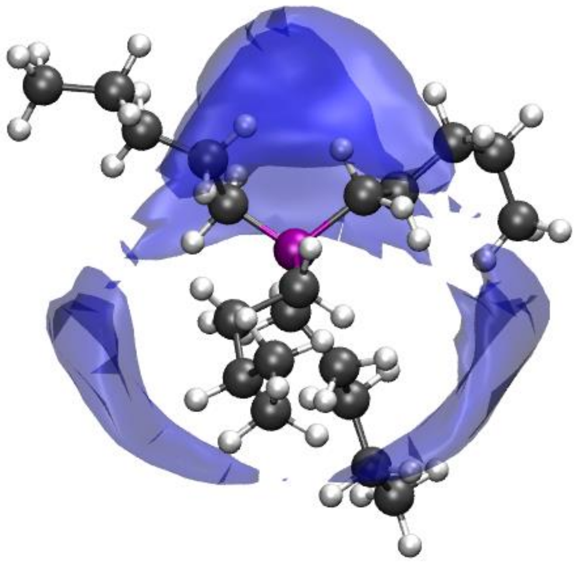

Figure S21: Spatial distribution function showing the coordination of anions around cations for [P5555][BF_4]. This isosurface is drawn at 50 mJ mol^{-1} K^{-1} Å^{-3}.

The well-defined structure in [P5555][BF_4] – compared to [P(3O1)$_4$][BF_4] – leave a signature in the (running integral of the) Coulomb interaction energy, Figure S22, see also our discussion in the preceding work.[23] Critically, the curling in ether functionalized ionic liquids leads to a longer oscillation period and shorter Debye length due to the stretched charge network and the more diffuse coordination, respectively. The difference in the charge network is also immediately visible in the static charge-charge correlation structure factor, Figure S23. This structure factor has been calculated using the center of charge of ions as described in the literature.[24] The characteristic charge-charge network distance is larger for [P(3O1)$_4$][BF_4] compared to [P5555][BF_4], the peak shift corresponds to 1 Å in real space.

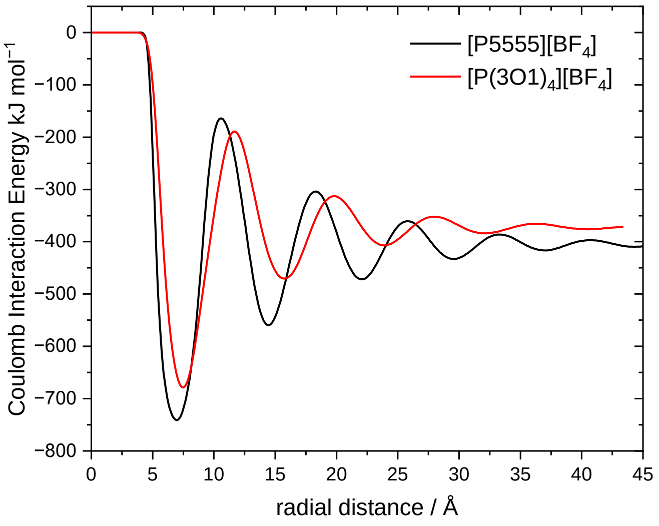

Figure S22: Coulomb interaction energy as a function of distance.

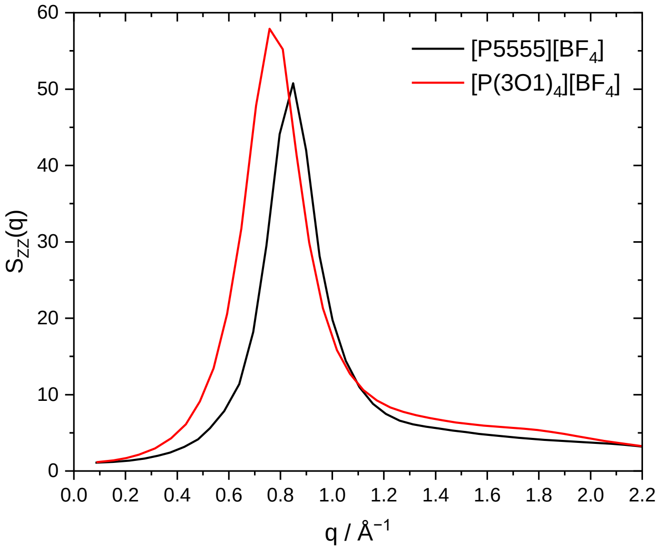

Figure S23: Static charge-charge correlation structure factor.

In this (atomistic) MD simulation, the curling of cations can also be assessed directly by calculating the backbone dihedral angles for each molecule at each point in the trajectory and binning the results into a histogram. Here, we use two-dimensional histograms with the same choice of dihedral angles as for the potential energy surfaces. For [P5555][BF_4], almost all cations are linear (not curled), Figure S24.

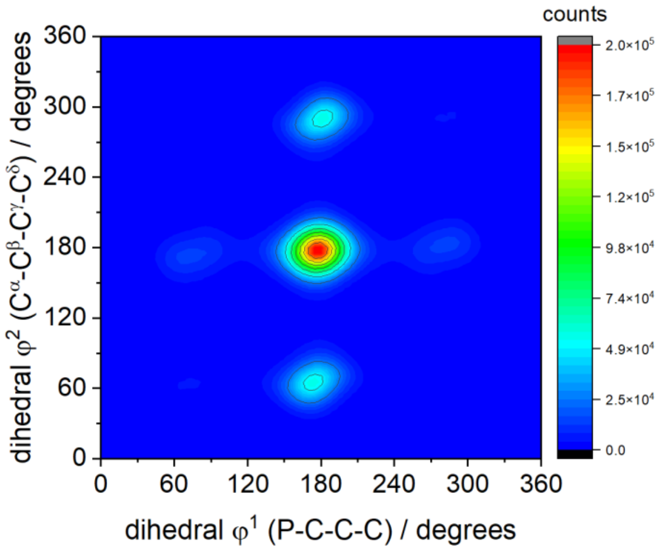

Figure S24: The population analysis of dihedral angles in the simulation of [P5555][BF_4] shows the expected strong preference for the linear conformations.

In contrast, for [P(3O1)$_4$][BF_4], virtually all cations assume one of the three curled conformers, Figure S25. Interestingly, a shift to conformer GS2 and GS3 can be observed. For better comparison, we also show the Boltzmann weighted potential energy surface obtained with the same force field, but for the [P111(3O1)]$^+$ model cation, Figure S26. Comparison of Figure S25 and Figure S26 shows that this shift is a many-body effect rather than a flaw of the force field, resulting either from interactions between the ether side chains or interactions with the anions.

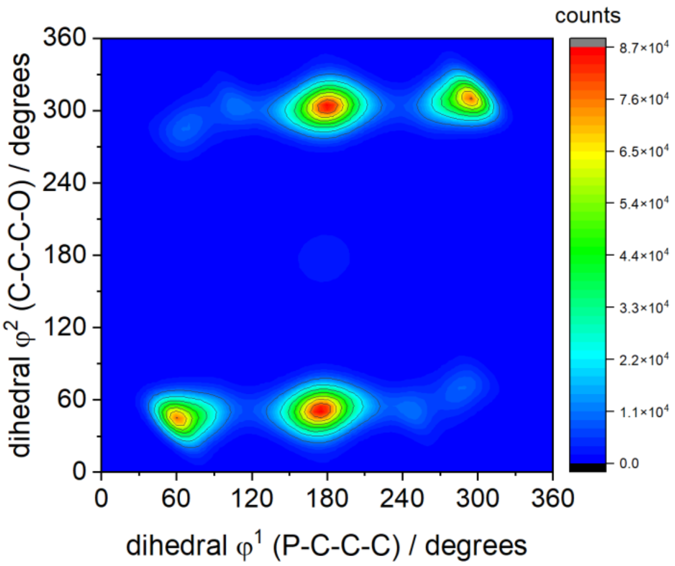

Figure S25: The population analysis of dihedral angles in the simulation of [P(3O1)$_4$][BF_4] shows the preference for GS2 and GS3, *i.e.* the curled conformers over linear ones.

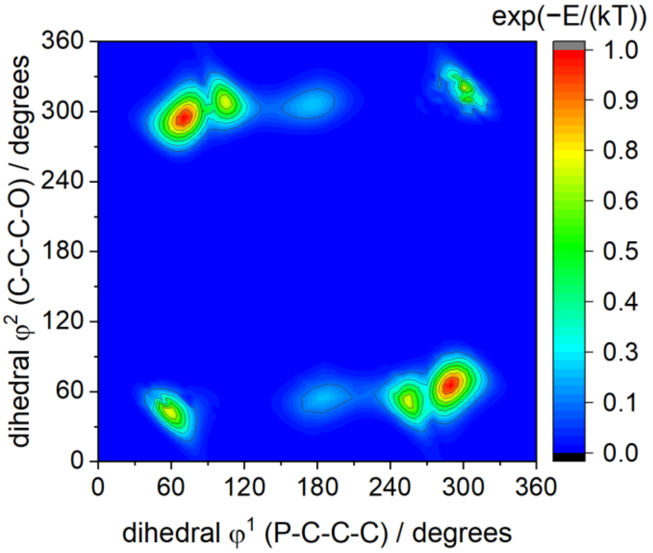

Figure S26: The Boltzmann-weighted potential energy surface of the small model cation for comparison with Figure S25. This surface was obtained from Figure S13 as exp(−E/(RT)) with T=400 K.

For the sake of completeness, we also briefly show dynamic properties obtained from the MD simulations. Here, only a qualitative comparison is possible due to the simulation setup (non-polarizable force field, 400 K). In general, the dynamics are significantly increased for the ether-functionalized ionic liquid, in line with our experimental observations. The charge network relaxation time can be obtained from the dynamic charge-charge correlation structure factor, Figure S27, with a tri-exponential fit as described previously.[24] Thus, lifetimes of 287 ps and 926 ps were obtained for $[P(3O1)_4][BF_4]$ and $[P5555][BF_4]$, respectively.

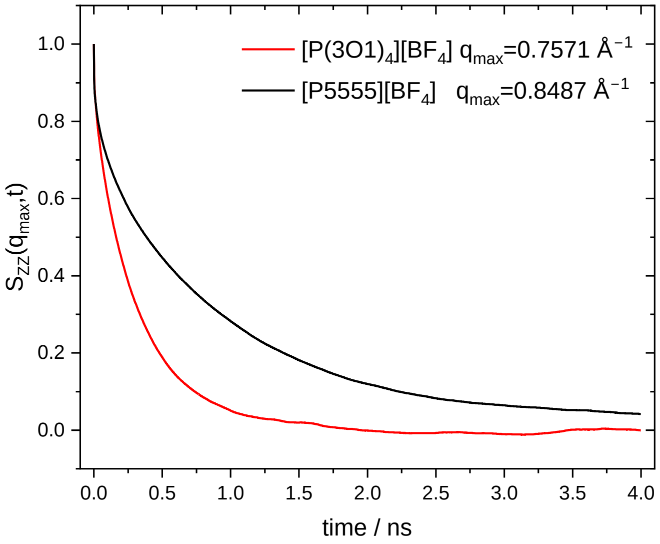

Figure S27: Dynamic charge-charge correlation structure factor, evaluated at the position of the peak maximum in Figure S23.

The separate dynamics of the cations and anions can be investigated using the mean squared displacement, Figure S28. The ballistic movement at short times is described by the mass of the ions, *i.e.* the anions show larger initial displacement than the cations. A crossover occurs around 30 ps, where the dynamics of cations and anions approach each other over longer timescales.

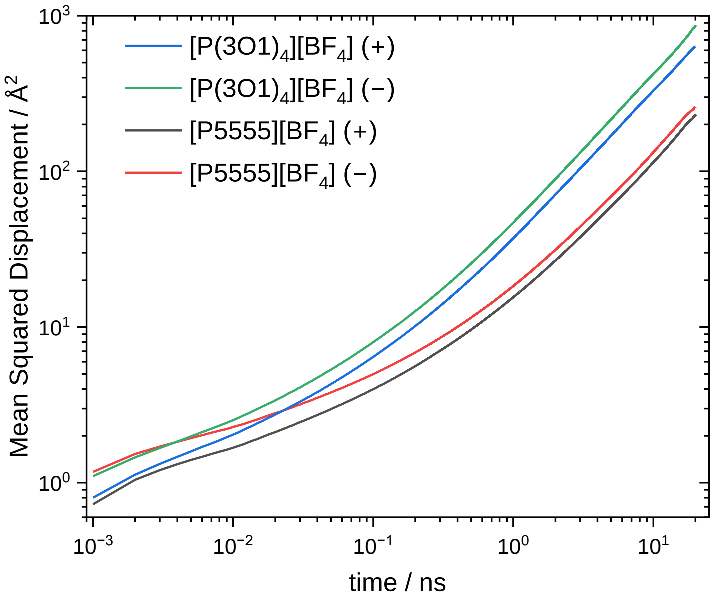

Figure S28: Mean squared displacements of the center of mass motions of cations (+) and anions (−).

The dynamical heterogeneity in the system can be probed conveniently using the non-gaussian parameter α_2, Figure S29. Here, by far the lowest dynamical heterogeneity is observed for the cations in $[P(3O1)_4][BF_4]$, *i.e.* the timescales of relaxation are more homogeneous spatially.

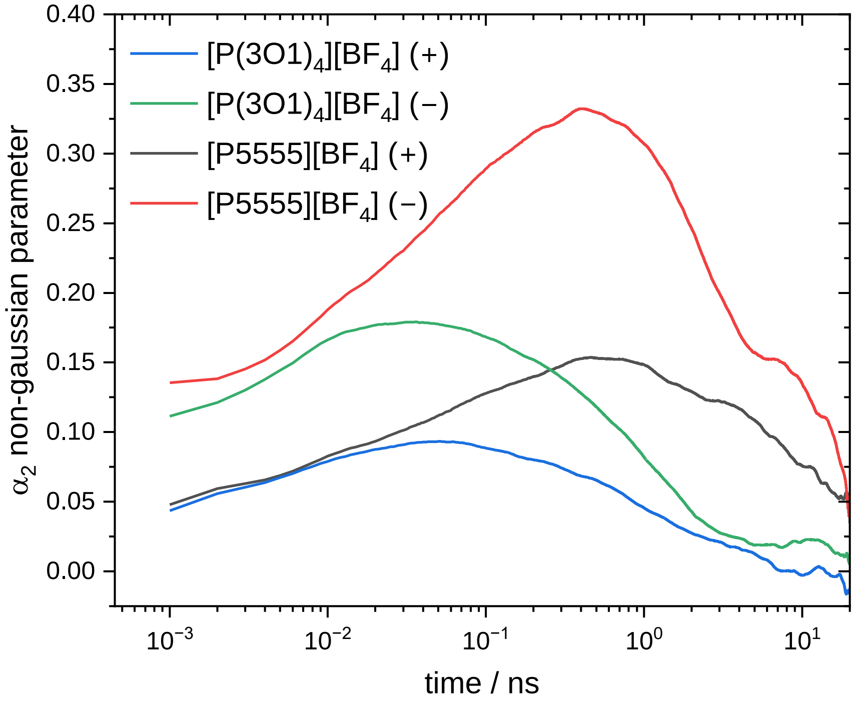

Figure S29: Non-gaussian parameter α_2 of the center of mass motions of cations (+) and anions (−).

5. Crystal Structure Analysis

The data for [P(3O1)$_4$]Br were collected using a Agilent Xcalibur 3 E diffractometer. The data for [P(3O1)$_4$][PF$_6$] were collected using a Bruker X8 Apex diffractometer. The data for [P5555]Br, [P5555][PF$_6$] and [P5555][BF$_4$] were collected using a Bruker D8 Venture diffractometer with a microfocus sealed tube and a Photon II detector. Graphite-monochromated Mo$_{Ka}$ radiation (λ = 0.71073 Å) was used. Data were collected at 153(2) K ([P(3O1)$_4$][PF$_6$]), 143(2) K ([P5555][PF$_6$], [P5555][BF$_4$] and [P5555]Br), or 173(2) K ([P(3O1)$_4$]Br) and corrected for absorption effects using the multi-scan method. The structure was solved by direct methods using SHELXT[25]) and was refined by full matrix least squares calculations on F^2 (SHELXL2018[26]) in the graphical user interface Shelxle[27]. See below and CCDC 2260785 to 2260790

5.1 Refinement details

All non H-atoms were located in the electron density maps and refined anisotropically. C-bound H atoms were placed in positions of optimized geometry and treated as riding atoms. Their isotropic displacement parameters were coupled to the corresponding carrier atoms by a factor of 1.2 (CH_2) or 1.5 (CH_3). *Disorder*: The $[PF_6]^-$ anion of [P5555][PF$_6$] is arranged on the intersection of the three twofold axes and was refined using the PART –1 instruction of SHELX. The $[BF_4]^-$ anion of [P5555][BF$_4$] is split over two positions about a crystallographic two-fold rotation axis and, hence a PART -1 and PART -2 instruction was used in the refinement process. The occupancy factors refined to 0.57 for the major component. In [P5555]Br three pentane-residues are split over two positions. Their occupancy factors refined to 0.72, 0.52, and 0.66 for the major components, respectively.

The absolute structure of [P(3O1)$_4$][Br] was determined by use of the Flack parameter [x = -0.020(9)]. Both the P1-based cation and the bromine anion were found to sit across independent C2 axes. For the former the axis passes through the phosphorus atom and bisects the C2···C2A and C7···C7A vectors, whilst for the latter the single atom sits on the axis.

5.2 Crystal Data and Structure Refinement

Table S14: Crystal data and structure refinement for $[P(3O1)_4][PF_6]$ and $[P5555][PF_6]$.

	$[P(3O1)_4][PF_6]$	$[P5555][PF_6]$
CCDC deposition number	2260785	2260786
Empirical formula	$C_{16}H_{36}F_6O_4P_2$	$C_{20}H_{44}F_6P_2$
Formula weight / g mol^{-1}	468.39	460.49
Temperature / K	154(2)	143(2)
Wavelength / Å	0.71073	0.71073
Crystal system	Monoclinic	Orthorhombic
Space group	$P2_1/n$	Ccca
Unit cell dimensions	a = 9.6155(2) Å	a = 11.1743(8) Å
	b = 15.4439(4) Å	b = 23.349(2) Å
	c = 15.4444(4) Å	c = 9.9798(6) Å
	α = 90°	α = 90°
	β = 93.6150(10)°	β = 90°
	γ = 90°	γ = 90°
Volume / Å^3	2288.94(10)	2603.8(3)
Z	4	4
Density (calculated) / g/cm^3	1.359	1.175
Absorption coefficient / mm^{-1}	0.256	0.213
F(000)	992	992
Crystal size / mm^3	0.200 x 0.200 x 0.020	0.300 x 0.180 x 0.010
Theta range for data collection	1.867 to 27.918°.	2.872 to 26.728°.
Index ranges	$-12 \le h \le 11$	$-14 \le h \le 11$
	$-19 \le k \le 20$	$-27 \le k \le 29$
	$-20 \le l \le 19$	$-12 \le l \le 12$
Reflections collected	22277	7677
Independent reflections	5485 [R(int) = 0.0407]	1382 [R(int) = 0.0562]
Completeness to theta = 25.242°	100.0%	99.6 %
Absorption correction	Semi-empirical from equivalents	
Max. and min. transmission	0.7456 and 0.7070	0.7455 and 0.6467
Refinement method	Full-matrix least-squares on F^2	
Data / restraints / parameters	5485 / 0 / 257	1382 / 145 / 113
Goodness-of-fit on F^2	1.017	1.053
Final R indices [I > 2sigma(I)]	R1 = 0.0388, wR2 = 0.0794	R1 = 0.0423, wR2 = 0.0889
R indices (all data)	R1 = 0.0630, wR2 = 0.0899	R1 = 0.0736, wR2 = 0.1018
Extinction coefficient	n/a	n/a
Largest diff. peak and hole	0.336 and –0.328 e Å^{-3}	0.181 and –0.199 e Å^{-3}

Table S15: Crystal data and structure refinement for [P5555][BF_4] and [P5555]Br.

	[P5555][BF_4]	[P5555]Br
CCDC deposition number	2260787	2260788
Empirical formula	$C_{20}H_{44}BF_4P$	$C_{20}H_{44}BrP$
Formula weight / g mol^{-1}	402.33	395.43
Temperature / K	143(2)	143(2)
Wavelength / Å	0.71073	0.71073
Crystal system	Orthorhombic	Monoclinic
Space group	Pcca	P21/c
Unit cell dimensions	a = 23.535(4) Å	a = 11.605(2) Å
	b = 10.8949(17) Å	b = 14.647(3) Å
	c = 9.5632(15) Å	c = 13.946(3) Å
	α = 90°	α = 90°
	β = 90°	β = 93.312(6)°
	γ = 90°	γ = 90°
Volume / Å^3	2452.1(7)	2369.9(8)
Z	4	4
Density (calculated) / g/cm^3	1.090	1.108
Absorption coefficient / mm^{-1}	0.144	1.800
F(000)	880	856
Crystal size / mm^3	0.400 x 0.400 x 0.040	0.210 x 0.160 x 0.060
Theta range for data collection	2.548 to 25.677°.	2.017 to 25.027°.
Index ranges	–28 ≤ h ≤ 28	–13 ≤ h ≤ 13
	–13 ≤ k ≤ 12	–17 ≤ k ≤ 15
	–11 ≤ l ≤ 11	–16 ≤ l ≤ 16
Reflections collected	19707	20644
Independent reflections	2289 [R(int) = 0.0702]	4158 [R(int) = 0.0815]
Completeness to theta = 25.242°	98.2 %	99.3 %
Absorption correction	Semi-empirical from equivalents	
Max. and min. transmission	0.7455 and 0.5928	0.7455 and 0.4829
Refinement method	Full-matrix least-squares on F^2	
Data / restraints / parameters	2289 / 66 / 176	4158 / 424 / 310
Goodness-of-fit on F^2	1.219	1.028
Final R indices [I > 2sigma(I)]	R1 = 0.0679, wR2 = 0.1367	R1 = 0.0598, wR2 = 0.1481
R indices (all data)	R1 = 0.0907, wR2 = 0.1476	R1 = 0.1077, wR2 = 0.1779
Extinction coefficient	n/a	n/a
Largest diff. peak and hole	0.192 and –0.349 e Å^{-3}	0.652 and –0.583 e Å^{-3}

Table S16: Crystal data and structure refinement for $[P(3O1)_4]Br$.

	$[P(3O1)_4]Br$
CCDC deposition number	2260790
Empirical formula	$C_{16}H_{36}BrO_4P$
Formula weight / g mol^{-1}	403.33
Temperature / K	173(2)
Wavelength / Å	0.71073
Crystal system	Monoclinic
Space group	C2
Unit cell dimensions	a = 22.6790(12) Å
	b = 5.6876(3) Å
	c = 8.1653(4) Å
	α = 90°
	β = 99.864(5)°
	γ = 90°
Volume / Å^3	1037.66(9)
Z	2
Density (calculated) / g/cm^3	1.291(9)
Absorption coefficient / mm^{-1}	2.071
F(000)	428
Crystal size / mm^3	0.300 x 0.100 x 0.070
Theta range for data collection	2.532 to 25.008°.
Index ranges	$-26 \le h \le 19$
	$-6 \le k \le 4$
	$-6 \le l \le 9$
Reflections collected	1722
Independent reflections	1309 [R(int) = 0.0118]
Completeness to theta = 25.242°	98.8 %
Absorption correction	Semi-empirical from equivalents
Max. and min. transmission	0.898 and 0.787
Refinement method	Full-matrix least-squares on F^2
Data / restraints / parameters	1309 / 1 / 103
Goodness-of-fit on F^2	1.045
Final R indices [I > 2sigma(I)]	R1 = 0.0205, wR2 = 0.0471
R indices (all data)	R1 = 0.0219, wR2 = 0.0479
Extinction coefficient	n/a
Largest diff. peak and hole	0.207 and –0.204 e Å^{-3}

Table S17: Torsion angles and P-O distance for the cation in the single crystal structure of $[P(3O1)_4][PF_6]$. Torsion angles are given when measuring from the phosphorous central atom along the chain with the starting carbon atom number.

Starting Carbon atom number	P-C_α-C_β-C_γ / °	C_α-C_β-C_γ-O / °	C_β-C_γ-O-C_ε / °	Distance P-O / Å
C1	–176.99(13)	–58.5(2)	–177.078(150)	4.5478(13)
C5	159.01(13)	–63.1(2)	–72.968(184)	4.4543(13)
C9	178.17(12)	–65.72(19)	178.456(145)	4.5614(14)
C13	–160.27(13)	60.0(2)	179.315(157)	4.3249(13)

Table S18: Torsion angles and distance of the central phosphorous atom for the cation in the single crystal structure of $[P5555][PF_6]$. Torsion angles are given when measuring from the phosphorous central atom along the chain.

P- C_α-C_β-C_γ / °	C_α-C_β-C_γ-C_δ / °	C_β-C_γ-C_δ-C_ε / °	Distance P-C_δ / Å
179.477(134)	177.204(165)	-175.466(179)	5.3473(22)

Table S19: Torsion angles and distance of the central phosphorous atom for the cation in the single crystal structure of $[P(3O1)_4][Br]$. Torsion angles are given when measuring from the phosphorous central atom along the chain with the starting carbon atom number.

Starting Carbon atom number	P-C_α-C_β-C_γ / °	C_α-C_β-C_γ-O / °	C_β-C_γ-O-C_ε / °	Distance P-O / Å
C1	–173.1(2)	62.0(4)	–175.342(288)	4.4547(28)
C5	–170.5(2)	61.9(3)	–171.920(312)	4.4461(25)

Table S20: Torsion angles and distance of the central phosphorous atom for the cation in the single crystal structure of [P5555][Br]. Torsion angles are given when measuring from the phosphorous central atom along the chain with the starting carbon atom number for the side chains with the highest side occupancy factors.

Starting Carbon atom number	P-C_α-C_β-C_γ / °	C_α-C_β-C_γ-C_δ / °	C_β-C_γ-C_δ-C_ε / °	Distance P-C_δ / Å
C1	177.624(314)	179.705(391)	179.705(391)	5.3251(52)
C6	–168.818(1187)	–170.487(1325)	–167.610(1453)	5.2889(121)
C9	161.305(679)	–73.212(1063)	–168.842(930)	4.6418(100)
C16	166.902(1373)	–138.875(1918)	–74.633(2239)	5.3521(176)

Table S21: Torsion angles and distance of the central phosphorous atom for the cation in the single crystal structure of [P(3O1)$_4$][BF$_4$]. Torsion angles are given when measuring from the phosphorous central atom along the chain with the starting carbon atom number.

Starting Carbon atom number	P-C_α-C_β-C_γ / °	C_α-C_β-C_γ-C_δ / °	C_β-C_γ-C_δ-C_ε / °	Distance P-C_δ / Å
C1	–179.736(198)	177.643(241)	178.802(249)	5.3349(33)
C6	–179.158(199)	–178.568(243)	176.854(265)	5.3556(33)

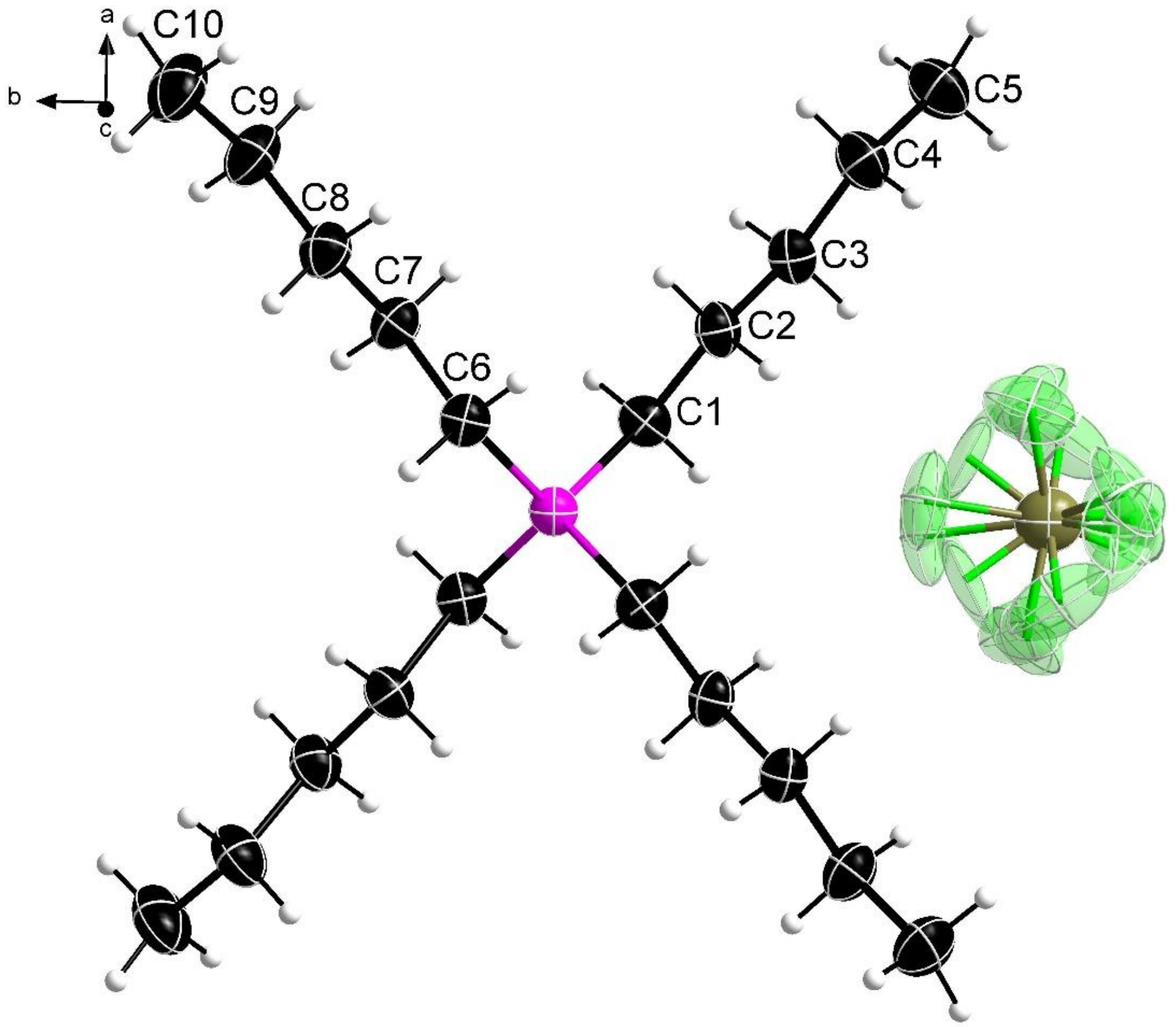

Figure S30: Single crystal structure of [P5555][BF$_4$].

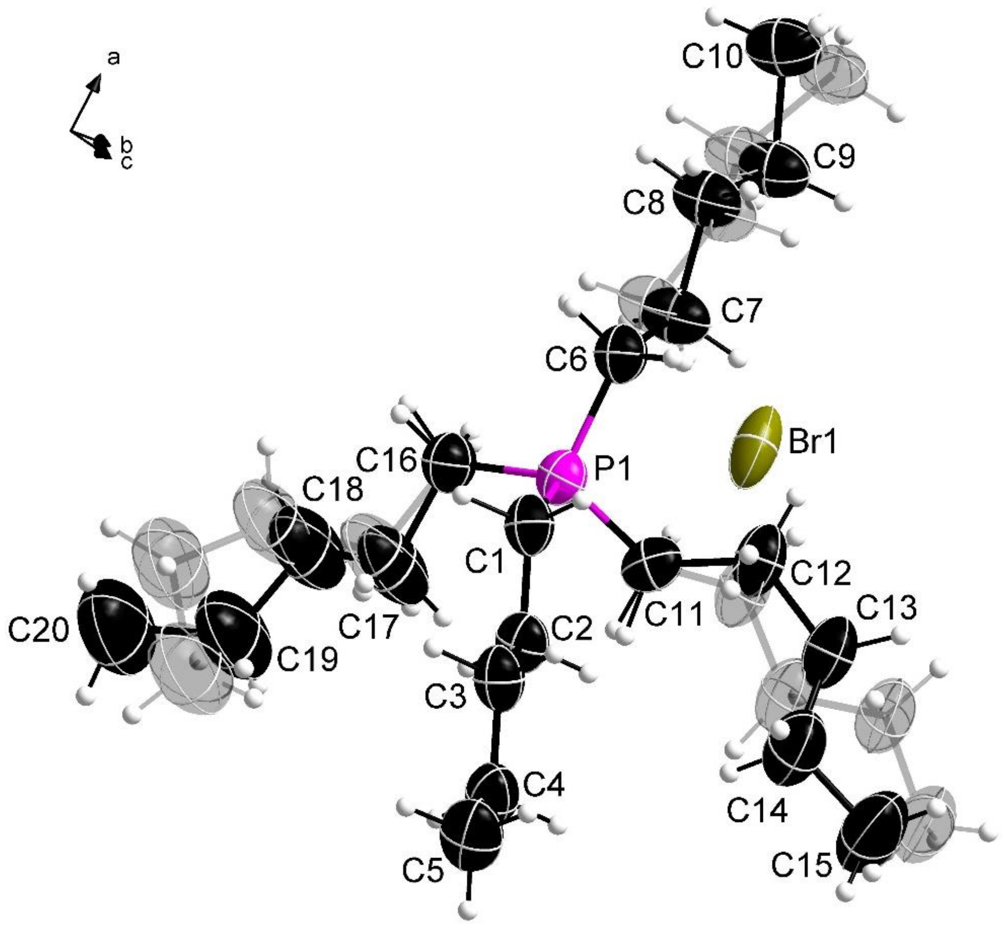

Figure 31: Single crystal structure of [P5555]Br.

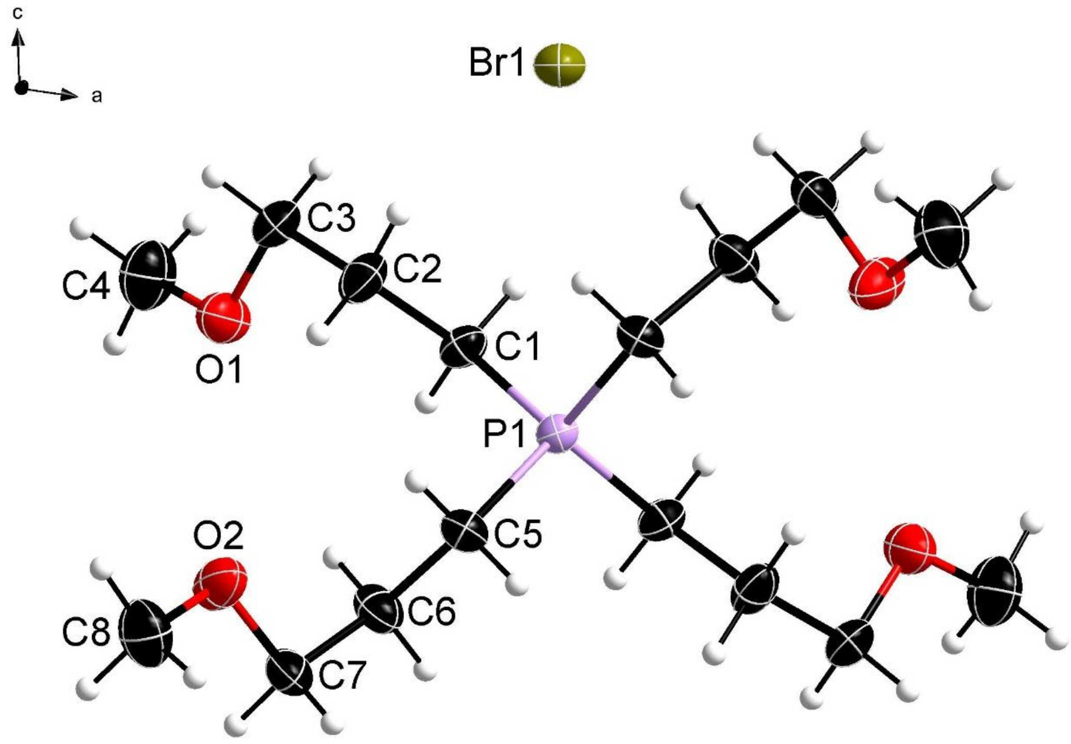

Figure 32: Single crystal structure of $[P(3O1)_4]Br$.

6. References

1 F. Herrmann and N. Kuhn, *Zeitschrift für Naturforsch. B*, 2012, **67**, 853–854.

2 F. Philippi, D. Rauber, B. Kuttich, T. Kraus, C. W. M. Kay, R. Hempelmann, P. A. Hunt and T. Welton, *Phys. Chem. Chem. Phys.*, 2020, **22**, 23038–23056.

3 F. Philippi, D. Rauber, J. Zapp, C. Präsang, D. Scheschkewitz and R. Hempelmann, *ChemPhysChem*, 2019, **20**, 443–455.

4 C. Ammann, P. Meier and A. Merbach, *J. Magn. Reson.*, 1982, **46**, 319–321.

5 Gaussian 09, Revision E.01, M. J. Frisch, G. W. Trucks, H. B. Schlegel, G. E. Scuseria, M. A. Robb, J. R. Cheeseman, G. Scalmani, V. Barone, B. Mennucci, G. A. Petersson, H. Nakatsuji, M. Caricato, X. Li, H. P. Hratchian, A. F. Izmaylov, J. Bloino, G. Zheng, J. L. Sonnenberg, M. Hada, M. Ehara, K. Toyota, R. Fukuda, J. Hasegawa, M. Ishida, T. Nakajima, Y. Honda, O. Kitao, H. Nakai, T. Vreven, J. A. Montgomery, Jr., J. E. Peralta, F. Ogliaro, M. Bearpark, J. J. Heyd, E. Brothers, K. N. Kudin, V. N. Staroverov, T. Keith, R. Kobayashi, J. Normand, K. Raghavachari, A. Rendell, J. C. Burant, S. S. Iyengar, J. Tomasi, M. Cossi, N. Rega, J. M. Millam, M. Klene, J. E. Knox, J. B. Cross, V. Bakken, C. Adamo, J. Jaramillo, R. Gomperts, R. E. Stratmann, O. Yazyev, A. J. Austin, R. Cammi, C. Pomelli, J. W. Ochterski, R. L. Martin, K. Morokuma, V. G. Zakrzewski, G. A. Voth, P. Salvador, J. J. Dannenberg, S. Dapprich, A. D. Daniels, O. Farkas, J. B. Foresman, J. V. Ortiz, J. Cioslowski, and D. J. Fox, Gaussian, Inc., Wallingford CT, **2013**.

6 S. Plimpton, *J. Comput. Phys.*, 1995, **117**, 1–19.

7 H. Sun, *J. Phys. Chem. B*, 1998, **102**, 7338–7364.

8 J.-P. Ryckaert, G. Ciccotti and H. J. C. Berendsen, *J. Comput. Phys.*, 1977, **23**, 327–341.

9 G. J. Martyna, D. J. Tobias and M. L. Klein, *J. Chem. Phys.*, 1994, **101**, 4177–4189.

10 M. Brehm and B. Kirchner, *J. Chem. Inf. Model.*, 2011, **51**, 2007–2023.

11 M. Brehm, M. Thomas, S. Gehrke and B. Kirchner, *J. Chem. Phys.*, 2020, **152**, 164105.

12 W. Humphrey, A. Dalke and K. Schulten, *J. Mol. Graph.*, 1996, **14**, 33–38.

13 T. LU and F. CHEN, *J. Theor. Comput. Chem.*, 2012, **11**, 163–183.

14 T. Lu and F. Chen, *J. Comput. Chem.*, 2012, **33**, 580–592.

15 J. N. Canongia Lopes and A. A. H. Pádua, *Theor. Chem. Acc.*, 2012, **131**, 1129.

16 J. N. Canongia Lopes, J. Deschamps and A. A. H. Pádua, *J. Phys. Chem. B*, 2004, **108**, 2038–2047.

17 J. N. Canongia Lopes and A. A. H. Pádua, *J. Phys. Chem. B*, 2006, **110**, 19586–19592.

18 W. L. Jorgensen, D. S. Maxwell and J. Tirado-Rives, *J. Am. Chem. Soc.*, 1996, **118**, 11225–11236.

19 G. Kaminski and W. L. Jorgensen, *J. Phys. Chem.*, 1996, **100**, 18010–18013.

20 J. B. B. Beckmann, D. Rauber, F. Philippi, K. Goloviznina, J. A. Ward-Williams, A. J. Sederman, M. D. Mantle, A. Pádua, C. W. M. Kay, T. Welton and L. F. Gladden, *J. Phys. Chem. B*, 2022, **126**, 7143–7158.

21 L. Martínez, R. Andrade, E. G. Birgin and J. M. Martínez, *J. Comput. Chem.*, 2009, **30**, 2157–2164.

22 A. Baranyai and D. J. Evans, *Phys. Rev. A*, 1989, **40**, 3817–3822.

23 D. Rauber, F. Philippi, B. Kuttich, J. Becker, T. Kraus, P. Hunt, T. Welton, R. Hempelmann and C.

W. M. Kay, *Phys. Chem. Chem. Phys.*, 2021, **23**, 21042–21064.

24 F. Philippi, D. Rauber, O. Palumbo, K. Goloviznina, J. McDaniel, D. Pugh, S. Suarez, C. C. Fraenza, A. Padua, C. W. M. Kay and T. Welton, *Chem. Sci.*, 2022, **13**, 9176–9190.

25 G. M. Sheldrick, *Acta Crystallogr. Sect. A Found. Adv.*, 2015, **71**, 3–8.

26 G. M. Sheldrick, *Acta Crystallogr. Sect. C Struct. Chem.*, 2015, **71**, 3–8.

27 C. B. Hübschle, G. M. Sheldrick and B. Dittrich, *J. Appl. Crystallogr.*, 2011, **44**, 1281–1284.
